# CIA5 and its interacting metal‐binding GTPase ZNG3 are degraded by the proteasome in Zn deficiency

**DOI:** 10.1111/tpj.71035

**Published:** 2026-07-14

**Authors:** George Kusi‐Appiah, Stefan Schmollinger, Andrew Mamo, Sarah C. Stainbrook, Thomas V. O'Halloran, Daniela Strenkert

**Affiliations:** ^1^ Department of Energy Plant Research Laboratory Michigan State University East Lansing Michigan 48824 USA; ^2^ Department of Biochemistry and Molecular Biology Michigan State University East Lansing Michigan 48824 USA; ^3^ Department of Physiology Michigan State University East Lansing 48824 Michigan USA; ^4^ Department of Chemistry Michigan State University East Lansing Michigan 48824 USA; ^5^ Department of Microbiology, Genetics and Immunology Michigan State University East Lansing Michigan 48824 USA; ^6^ Department of Plant Biology Michigan State University East Lansing Michigan 48824 USA

**Keywords:** metallochaperone, COG0523, pyrenoid, CCM1, CBP1

## Abstract

Carbon and zinc (Zn) metabolism are tightly connected in phototrophs, as carbonic anhydrases critical for CO_2_ assimilation are highly abundant Zn enzymes. The eukaryotic green alga *Chlamydomonas* (*Chlamydomonas reinhardtii*) maintains efficient phototrophic growth in ambient or low CO_2_ environments by establishing a carbon concentrating mechanism (CCM). We show that *Chlamydomonas* raises its cellular Zn quota to accommodate higher Zn demand under low CO_2_ conditions, an adjustment that is dependent on CIA5, a major regulator of the CCM. We demonstrate that *CIA5* is constitutively expressed regardless of Zn supply, but its encoded protein undergoes proteasomal degradation under Zn deficiency, providing a checkpoint that prevents CCM induction when Zn cofactors are unavailable. We identified a COBW domain‐containing GTPase, ZNG3, as a constitutive interacting partner of CIA5. As CIA5, ZNG3 does not accumulate in low Zn and is degraded by the proteasome. Unlike *cia5* mutants, *zng3* mutants grow like wild‐type without CO_2_ supplementation but exhibit a growth defect when grown in the presence of high CO_2_ or acetate. Transcriptome sequencing revealed that expression of genes encoding central components of the CCM is mostly unchanged in *zng3* mutants, while a subset of genes induced in Zn deficiency, including *ZRT2* encoding for a Zn transporter, is increasingly expressed. Under low CO_2_ conditions, *cia5 zng3* double mutants exhibit a more severe phenotype than *cia5* single mutants. This result points to a condition‐dependent genetic interaction between *CIA5* and *ZNG3*, suggesting both proteins provide distinct but partially overlapping contributions to fitness depending on carbon availability.

## Significance Statement

Phototrophs must balance carbon and zinc metabolism to account for higher zinc demand under low CO_2_ supply, since increased amounts of abundant Zn proteins (carbonic anhydrases) are required for the CCM in green algae. We identified a regulatory bipartite module in the eukaryotic green alga *Chlamydomonas reinhardtii*, consisting of the Zn finger protein CIA5 and the metal‐binding GTPase ZNG3.

## INTRODUCTION

Algae are crucial primary producers, accounting for approximately 50% of all photosynthetic carbon fixation on Earth (Field et al., 1998; Gerotto et al., 2020), and are equally important model organisms for fundamental research in photosynthesis and nutrient metabolism (Salomé & Merchant, 2019). Algae thrive in diverse ecological niches not occupied for agriculture, even in environments that are characterized by extreme conditions (de Vargas et al., 2015), demonstrating substantial metabolic flexibility and adaptability (Varshney et al., 2015).

Many algae, including the tractable, eukaryotic model *Chlamydomonas* (*Chlamydomonas reinhardtii*), utilize carbon concentrating mechanisms (CCMs) to grow efficiently in their naturally low CO_2_ habitats (Badger & Price, 2003; Giordano et al., 2005; Roberts et al., 2007; Wang et al., 2011). The CCM facilitates the uptake of both bicarbonate (HCO_3_^−^) and CO_2_ against a gradient, achieving approximately 10–40‐fold higher inorganic carbon (C_i_) concentrations within cells than in the environment (Badger et al., 1980). CO_2_ and HCO_3_^−^ transporter mediate import into cells or organelles (Wang et al., 2011), while carbonic anhydrases (CAHs) facilitate the interconversion between CO_2_ and HCO_3_^−^ to aid in transport or provide CO_2_ for carbon fixation (Jordan & Ogren, 1981; Moroney & Ynalvez, 2007; Wang et al., 2015). CO_2_ is ultimately assimilated by Ribulose‐1,5‐bisphosphate (RuBP) carboxylase oxygenase (RuBisCO), at the center of the Calvin–Benson–Bassham (CBB) cycle in the chloroplast, converting RuBP into two molecules of 3‐phosphoglycerate (3PGA) (Benson et al., 1952). RuBisCO can also catalyze the oxygenation of RuBP, at a rate that depends on temperature, the CO_2_:O_2_ ratio, and inherent enzymatic properties. Under ambient conditions, three CO_2_ molecules are fixed for one O_2_ reacted with RuBP (Griffiths, 2006). RuBP oxygenation produces one 2‐phosphoglycolate (2PG) molecule instead of 3PGA, which is recycled via energetically costly photorespiration to minimize the loss of carbon backbones (Bauwe et al., 2010). RuBisCO selectivity for CO_2_ over O_2_ can be improved by raising the CO_2_ concentration and minimizing O_2_ exposure around the enzyme. For this purpose, eukaryotic algae such as *Chlamydomonas* use pyrenoids (Goodenough & Levine, 1970; Wang et al., 2015); self‐assembling, membrane‐less structures that form starch sheaths to create a diffusion barrier around RuBisCO (Mackinder et al., 2016; Toyokawa et al., 2020). Similar strategies are present in diatoms that use protein‐walled pyrenoids (Shimakawa et al., 2024), while cyanobacteria fix CO_2_ in protein microcompartments called carboxysomes (Nguyen et al., 2025; Turmo et al., 2017). On land, plants have evolved C_4_ photosynthesis (Sage, 2004) and Crassulacean acid metabolism (CAM) (Griffiths, 1989) to minimize CO_2_ loss during photosynthetic carbon fixation (van Lun et al., 2014).

The CCM components do not accumulate under all conditions in *Chlamydomonas*, but instead are dynamically induced when cells are exposed to very low level (<0.01%), or air level of CO_2_ (0.04%) during growth (Badger et al., 1980). A forward genetic screen identified a major regulator of the CCM, the constitutively expressed gene *Carbon Inorganic Accumulation 5* (*CIA5*, also reported as *CCM1*) (*CIA5* hereafter) (Fukuzawa et al., 2001; Moroney et al., 1989; Xiang et al., 2001). CIA5 localizes to the nucleus, and *cia5* mutants fail to transcriptionally induce C_i_ transporter genes, *CAH*s, and many genes encoding structural components of the CCM; indeed, *cia5* mutants have a growth defect under low CO_2_ supply (Fang et al., 2012; Fukuzawa et al., 2001; Wang et al., 2005; Xiang et al., 2001). Transcriptome deep sequencing (RNA‐seq) studies identified approximately 3500 low‐CO_2_‐inducible (*LCI*) genes whose expression partially or fully fail to respond to low CO_2_ conditions in *cia5* mutants, indicating a wider restructuring of *Chlamydomonas* metabolism in CO_2_‐limiting environments (Brueggeman et al., 2012; Fang et al., 2012). Complementation studies identified two regions critical for CIA5 function: approximately 110 amino acids at the N terminus and approximately 130 amino acids toward the C terminus were sufficient to restore growth under very low CO_2_ conditions and partially restored molecular phenotypes to wild‐type (wt) levels (Moroney & Ynalvez, 2007). A confirmed transcriptional regulator, LCR1, which acts downstream of CIA5, was shown to regulate a subset of genes involved in low CO_2_ acclimation (Yoshioka et al., 2004). The chloroplast‐localized calcium (Ca^2+^)‐binding protein CAS is also involved in maintaining expression of LCI genes in *Chlamydomonas* (Wang et al., 2016).

It remains unclear mechanistically how CIA5 is activated to induce the *Chlamydomonas* CCM, but previous work highlighted the dependence of successful acclimation to low CO_2_ environments on zinc (Zn) availability (Hsieh et al., 2013; Malasarn et al., 2013). Notably, approximately 4–10% of all proteins in eukaryotes are estimated to utilize Zn as an essential cofactor (Andreini et al., 2006a, 2006b). While over 400 eukaryotic enzymes depend upon Zn in their active site to mediate catalysis, most Zn‐dependent proteins rely on Zn to stabilize their structure, most prominently in Zn finger domains involved in interactions with DNA, RNA, or proteins (Andreini et al., 2006a, 2006b; Dupont et al., 2010; Han et al., 2020). One such example is CIA5, which contains two Zn‐binding sites within the first 110 amino acids, one in a C_2_H_2_ zinc finger between amino acids 35 and 70, and the second between amino acids 70 and 100 (Andreini et al., 2006a; Dupont et al., 2010; Kohinata et al., 2008). In fact, the first isolated *cia5* mutant harbored a mutation responsible for a single amino acid change replacing histidine residue H54 in the first Zn‐binding site to a tyrosine residue (Fukuzawa et al., 2001; Moroney et al., 1989; Xiang et al., 2001). Chloroplast‐localized BUNDLE SHEATH DEFECTIVE 2 (BSD2) is another Zn finger protein requiring two Zn atoms for its function (Aigner et al., 2017). BSD2 is a small, RuBisCO‐specific chaperone involved in the assembly of the L_8_S_8_ RuBisCO hexadecamer and is thus required to allow CO_2_ fixation (Aigner et al., 2017; Brutnell et al., 1999; Wang et al., 2023). CAHs are abundant and integral enzymes of CCMs; they are present in all cellular compartments and rely on Zn atoms in their catalytic center for the reversible hydration reaction of CO_2_ to HCO_3_^−^ (Kupriyanova et al., 2023; Lindskog & Coleman, 1973). In marine habitats, where Zn can be scarce (Bruland, 1989), cobalt (Co) can replace Zn in the catalytic centers of some CAHs; in a few organisms, backup CAHs that rely on cadmium (Cd) can be used instead (Lane & Morel, 2000a). That such Zn‐sparing mechanisms evolved underscores the crucial contribution of CAHs and Zn to the phototrophic lifestyle (Lane & Morel, 2000b; Yee & Francois, 1996).

Transporters and shuttles involved in Zn import and distribution have been identified in *Chlamydomonas* using comparative RNA‐seq and proteomics analysis in Zn‐replete and low‐Zn environments, but it is unclear how Zn homeostasis is integrated into larger cellular regulatory networks (Hong‐Hermesdorf et al., 2014; Hsieh et al., 2013; Strenkert et al., 2023). Most prokaryotic and eukaryotic genomes contain one and often more genes that encode CobW/COG0523 proteins, defining a conserved family of metallochaperones (Haas et al., 2009). CobW/COG0523 proteins are themselves members of the larger G3E GTPase superfamily, which contains metallochaperones able to handle nickel (Ni) or Co (HypB, UreG, MeaB) (Haas et al., 2009; Leipe et al., 2002). The first protein identified from the COG0523 subgroup of GTPases was CobW, which is involved in cobalamin (Vitamin B_12_) biosynthesis, likely by delivering Co for insertion into the corrin ring (Crouzet et al., 1991; Haas et al., 2009; Young et al., 2021). CobW‐like proteins can deliver various metals to individual clients, including iron (Fe), Zn, and Co (Blaby‐Haas et al., 2012; Gumataotao et al., 2017; Jordan et al., 2019), and potentially Ni, although Zn appears to be their most common ligand (Haas et al., 2009). The specific metal delivered by individual CobW/COG0523 members is not easily inferred from primary sequences or *in vitro* affinities; instead, it is dependent on the physiological metal‐ion concentrations within the protein's native compartment. Client–protein association is commonly used to predict potential ligands (Haas et al., 2009; Young et al., 2021). Proteins belonging to the subfamily of Zn‐regulated GTPases (ZNGs) like ZNG1 have been identified as candidate Zn chaperones that can transfer Zn to Zn‐containing proteins based on domain structures, Zn‐binding capabilities, and GTPase activity (Pasquini et al., 2022). While the vertebrate protein ZNG1 was shown to interact with its clients via their Zn finger domain, Zn transfer appears to involve another Zn‐binding site within the client proteins (Weiss et al., 2022).

Here, we explored the role of Zn in the CCM of *Chlamydomonas* in more detail, with a focus on regulatory circuits, by using CIA5 as an entry point and by identifying potential molecular connections between the CCM and Zn metabolism.

## RESULTS

### CCM induction requires a substantial increase in the cellular Zn quota

**Figure 1:**
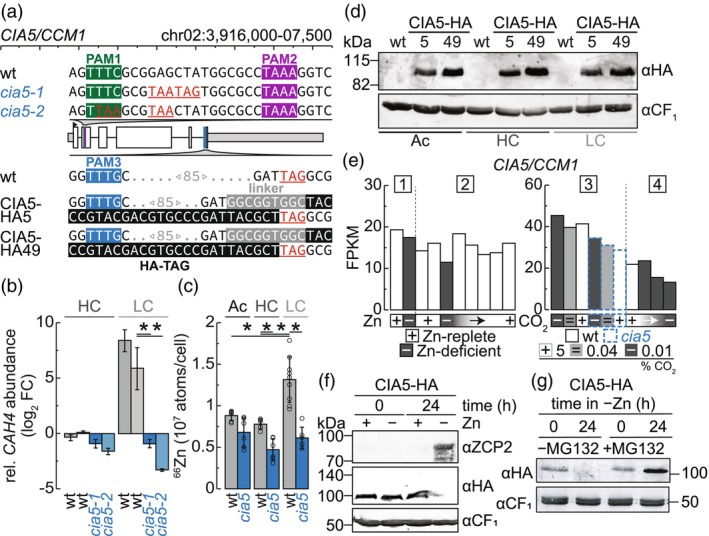
The Chlamydomonas zinc quota is adjusted based on carbon source in a Cia5‐dependent manner. (a) Diagram of the *Cia5* locus showing the palindrome‐adjacent motifs (PAMs) and the target sites used to generate knockout mutant strains (green and magenta) or HA knock‐in strains (blue) via CRISPR‐mediated gene editing. Stop codons were introduced in‐frame in the *cia5‐1* and *cia5‐2* mutants and are highlighted in red font; a short linker (gray) was added upstream of the sequence encoding the HA epitope (black) introduced before the Cia5 stop codon (red). (b) Relative *CAH4* transcript levels, determined by RT‐qPCR in wild‐type (wt) and *cia5* mutants grown phototrophically via bubbling with 5% CO_2_ (high CO_2_, HC) or 0.04% CO_2_ (low CO_2_, LC). Data are means ± standard deviation (SD) from three independent experiments and plotted as log_2_ fold‐change values compared with mean wt values under HC. Asterisks indicate significant differences (two‐sided *t*‐test, *P* < 0.05). (c) Zinc (Zn) content of wt and *cia5* mutant cells grown phototrophically under HC or LC, or photoheterotrophically (with acetate [Ac] as sole reduced carbon source), determined by ICP–MS/MS and normalized to cell number. Data are means ± SD from 3 to 9 independent experiments; individual data points are shown as open circles. Asterisks indicate significance (two‐sided *t*‐test, *P* < 0.05). (d) Immunoblot analysis of Cia5‐HA in wt and *CIA5‐HA* strains. Total protein was extracted from wt and two *Cia5‐HA* strains grown under Ac, HC, or LC conditions. Immunoblotting was performed with anti‐HA antibodies; CF_1_ served as loading control. (e) Survey of *CIA5* transcript abundance in four published RNA‐seq datasets of *Chlamydomonas* cells under varying Zn and CO_2_ supply: (1) Zn‐replete (+) and Zn‐deficient (−) cultures from Malasarn et al. (2013); (2) early exponential and early stationary Zn‐replete cultures (+), Zn‐deficient (−) cultures, and during Zn resupply (−➛+) from Hong‐Hermesdorf et al. (2014); (3) cultures acclimated to high (+, 5%), air‐level (=, 0.04%), or low CO_2_ (−, 0.01%) in wt (black outline) and a *cia5* mutant (blue, dashed outline) from Fang *et al*. (2012); (4) during the transition (+➛−) from high (5%) to very low CO_2_ supply (0.01%) from Brueggeman *et al*. (2012). (f) Immunoblot analysis of CIA5‐HA abundance as a function of Zn supply. Total protein was extracted from *CIA5‐HA5* strain grown photoheterotrophic with (+) or without (−) Zn for the indicated time. Immunoblotting was performed with anti‐HA antibodies; CF_1_ served as loading control, and ZCP2 served as control for successful establishment of Zn deficiency. (g) CIA5 is degraded by the proteasome under Zn‐deficient conditions. *CIA5‐HA*5 was subjected to Zn‐replete (+) or Zn‐deficient (−) conditions alone or with the addition of the proteasome inhibitor MG132. CF_1_ served as loading control. Shown is one of two independent experiments.

The ability of *Chlamydomonas* cells to engage the CCM in low CO_2_ environments and maintain a high phototrophic growth rate is impaired under Zn‐limiting conditions (Malasarn et al., 2013). This observation suggests that one or more Zn‐dependent components of the CCM are essential for acclimation to low CO_2_, with abundant Zn‐containing proteins like CAHs being likely candidates. In agreement with this idea, CAH abundance was very low in Zn‐deficient cells, and the poor phototrophic growth of Zn‐deficient cells could be rescued by bubbling with 1% CO_2_, a condition under which some of the CAHs and other components of the CCM are not needed (Malasarn et al., 2013). We therefore wondered if the Zn content of cells might change depending on the growth conditions, namely, photoheterotrophically with the addition of acetate as sole reduced carbon source, or phototrophically in reduced carbon‐free medium and bubbling with low CO_2_ (air level of 0.04% [v/v], LC) or high CO_2_ (5% [v/v], HC). Since *CAH* expression is dependent on the regulator CIA5, we generated new *cia5* mutants in the CC‐425 strain background, using a previously established protocol for Cpf1‐mediated gene editing (Ferenczi et al., 2017; Strenkert et al., 2024) to introduce two in‐frame stop codons into the first exon of *CIA5*. We obtained two independent *cia5* mutant strains (*cia5‐1* and *cia5‐2*) carrying two stop codons at the intended positions (Table S1, Figure 1a). We confirmed the loss of CIA5 function by probing the expression of *CAH4*, a known target of CIA5 encoding a mitochondrial β‐carbonic anhydrase, and by monitoring growth under phototrophic conditions with air‐level CO_2_ bubbling or high CO_2_ bubbling (Fang et al., 2012; Moroney et al., 2011; Rai et al., 2021). *CAH4* transcript levels were highly induced in wt cells in LC relative to HC, but not in the two *cia5* mutants (Figure 1b). *CAH4* was expressed at much lower levels in wt and the *cia5* mutants in HC, consistent with previous reports (Fang et al., 2012). We then determined the Zn content of wt and *cia5* mutant cells grown phototrophically (HC, LC) and photoheterotrophically by inductively coupled plasma–tandem mass spectrometry (ICP‐MS/MS). The Zn quota of LC‐grown wt cells rose by approximately 70% (*P* < 0.05) compared with HC cultures, and by approximately 50% (*P* < 0.05) compared with photoheterotrophic cultures (Figure 1c). This higher Zn content under LC conditions was not observed for the *cia5* mutants, consistent with a lack of acclimation to a low CO_2_ environment in these mutants and low *CAH* expression (Figure 1b,c). There was no significant difference in the cellular quotas of other metal cofactors Fe and Mn in LC between wt and the *cia5* mutants (Figure S1). Zn content was also lower in the *cia5* mutant cells than in wt cells under HC conditions, although not as markedly as under LC conditions; while Zn content was comparable among genotypes in cells grown photoheterotrophically. Mn content was only lower in the *cia5* mutants under HC conditions (Figure 1c, Figure S1).

The higher Zn quota seen in LC‐grown wt cells likely reflects the additional requirement for this metal imposed by highly abundant Zn‐containing proteins that are required for growth under this condition. Since this greater Zn demand depends on CIA5, we hypothesize that the various CAHs encoded by CIA5‐induced genes in LC contribute to this increased demand. These results also highlight the consequences of carbon availability on Zn metabolism and suggest that the higher Zn quota of *Chlamydomonas* cells during CCM establishment requires adjustments to Zn metabolism, including induction of genes encoding Zn importers and Zn‐handling proteins.

### Constitutive CIA5 abundance is Zn‐dependent

To assess CIA5 protein abundance and identify its potential interacting proteins, we introduced a sequence encoding a HA‐tag preceded by a small, flexible, glycine linker just upstream of the CIA5 stop codon in the native locus, using the CRISPR/Cpf1 gene editing approach (Figure 1a). We identified two independent strains, *CIA5‐HA5* and *CIA5‐HA49*, that accumulate the CIA5‐HA fusion protein from the endogenous locus, with no unintended mutations or frameshifts in the *CIA5* locus (Figure 1a,d). These knock‐in strains retain the normal regulation of CIA5 and only contain HA‐tagged CIA5. We chose a commercial anti‐HA antibody that showed no non‐specific cross‐reactivity against total *Chlamydomonas* cell lysates for immunoblot analysis of CIA5‐HA in cells grown phototrophically (HC, LC) or photoheterotrophically (Ac) in Zn‐replete medium. In agreement with earlier reports (Xiang et al., 2001), CIA5 abundance was constant regardless of growth conditions (Figure 1d). Based on its constant levels and nuclear localization independent of CO_2_ availability (Fang et al., 2012; Fukuzawa et al., 2001; Wang et al., 2005; Xiang et al., 2001), CIA5 function was previously proposed to be modulated by post‐translational modifications in establishing the CCM. There was no noticeable shift in the mobility of CIA5‐HA across the different growth conditions, and CIA5‐HA migrated as a single band; however, the apparent molecular weight of the fusion protein was higher in SDS‐PAGE than its predicted mass (~71.3 kDa + ~1.3 kDa from glycine linker and HA‐Tag), all findings consistent with previous results (Figure 1d; Wang et al., 2005).

When we surveyed *CIA5* transcript levels in several published RNA‐seq datasets from cells grown under different Zn supply and/or CO_2_ levels, we noticed no clear directional change in transcript abundance (Brueggeman et al., 2012; Fang et al., 2012; Hong‐Hermesdorf et al., 2014; Malasarn et al., 2013). *CIA5* transcript abundance did not show a significant increase or decrease in response to changes in Zn or carbon source in any of these experiments, aside from a transient rise in *CIA5* transcripts upon Zn resupply to Zn‐deficient cultures in the Hong‐Hermesdorf et al. (2014). dataset (Figure 1e). Since CIA5 itself is a Zn‐containing protein, we checked CIA5‐HA protein abundance under Zn‐replete and Zn‐deficient conditions in the *CIA5‐HA* strains, using cultures grown in TAP with full Zn supply only or shifted to TAP medium lacking Zn for 24 h (Figure 1f). We detected CIA5‐HA in cells experiencing Zn‐replete conditions, but not under Zn limitation (Figure 1f). As a control for Zn deficiency, we performed an immunoblot for ZCP2, a predicted but uncharacterized Zn chaperone encoded by a gene strictly induced in the absence of Zn (Hsieh et al., 2013; Malasarn et al., 2013). Indeed, ZCP2 accumulated only in cells grown in medium lacking Zn (Figure 1f). The observed loss of CIA5‐HA signal in Zn‐deficient cells suggested that CIA5 may be a substrate for degradation by a specific protease or the ubiquitin–proteasome system (UPS). We therefore tested the effect of treatment with MG‐132, a potent 26S proteasome inhibitor. CIA5‐HA protein abundance remained high following the addition of MG‐132 concomitantly with transfer to Zn‐deficient medium, suggesting that CIA5 is targeted for degradation by the UPS (Figure 1g).

Given that *CIA5* is constitutively transcribed independently of CO_2_ or Zn supply, it was surprising that CIA5 would accumulate under Zn‐replete but not Zn‐deficient growth conditions. These results highlight the dependence of CCM induction on Zn availability, dictated by the stability or translational control of the major regulator CIA5.

### CIA5 constitutively interacts with a metal‐binding GTPase

**Figure 2:**
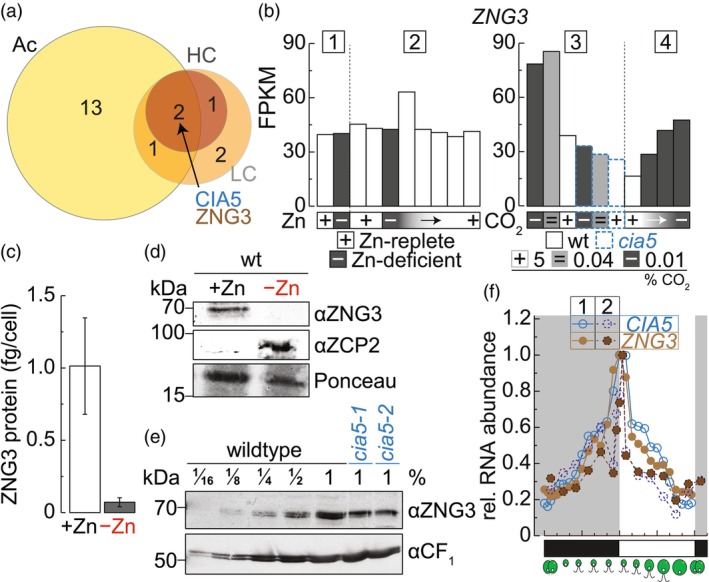
CIA5 constitutively interacts with ZNG3. (a) Venn diagram showing the extent of overlap among proteins identified as Cia5 interactors. Total protein from *Cia5‐HA* strains grown photoheterotrophically (Ac) or phototrophically (5% CO_2_ [HC] or 0.04% CO_2_ [LC]) was subjected to immunoprecipitation with anti‐HA antibodies; bound proteins were analyzed by LC–MS/MS. Only the number of identified proteins in the *CIA5‐HA* strains and not in wt controls is shown. (b) Survey of *ZNG3* transcript abundance in RNA‐seq datasets with varying Zn and CO_2_ supply, as described in Figure 1c. (c) Absolute protein abundance of ZNG3 under Zn‐replete (+Zn) or Zn‐deficient (−Zn) conditions, from published MS data (Strenkert et al., 2023). (d) Immunoblot analysis of ZNG3 under varying Zn supply. Total protein was extracted from wt cultures grown under Zn‐replete (+Zn) or Zn‐deficient (−Zn) conditions and analyzed as described in Figure 1d. Immunoblotting was performed with anti‐ZNG3 antibodies generated in this study. CF_1_ served as loading control, ZCP2 served as control for successful establishment of Zn deficiency. (e) Quantification of ZNG3 protein abundance in wt and the two *cia5* mutants. Total protein was extracted from wt, *cia5‐1* and *cia5‐2* grown under +Zn conditions and analyzed as described in Figure 1d. Wt samples were serially diluted to establish a dynamic range. Immunoblotting was performed with anti‐ZNG3 antibodies. CF_1_ served as loading control. (f) Survey of transcript abundance for *Cia5* and *ZNG3* in cells grown phototrophically under a 12‐h light/12‐h dark diurnal cycle, from published RNA‐seq datasets. (1) RNA abundance centered around “lights on” with approximately 2‐h resolution from Strenkert et al. (2019); (2) RNA abundance centered around “lights off” with approximately 1‐h resolution from Zones et al. (2015).

To explore the mechanism by which CIA5 establishes the CCM, we performed immunoprecipitations (IP) on cell lysates from the two independent *Cia5‐HA* strains grown under LC, HC, or Ac conditions, alongside lysates from wt cells as a negative control to account for non‐specific contaminants. We identified bound proteins by liquid chromatography–tandem mass spectrometry (LC‐MS/MS) using the *Chlamydomonas* v6 genome as a reference (Craig et al., 2023). We only considered proteins as being high‐confidence CIA5‐interacting partners if they were identified as two unique peptides or more and in IPs from both *CIA5‐HA5* and *CIA5‐HA49* but not in wt samples under any condition, yielding 3, 6, and 16 proteins that met these criteria in HC, LC, and Ac, respectively (Data S1, Figure 2a). Only two proteins were shared among all three conditions and were far more abundant than any other coprecipitated protein, based on precursor ion intensity (Data S1). The first protein was CIA5‐HA itself, validating the IPs. The second protein was a protein of unknown function, containing a nuclear localization signal (NLS, Figure S2) and a domain belonging to a multiprotein family of candidate metal‐handling GTPases (encoded by Cre16.g684650, hereafter designated as ZNG3).

### ZNG3 belongs to a conserved class of metal‐binding GTPases

Phylogenetic analysis revealed that ZNG3 belongs to the G3E family of P‐loop GTPases, specifically the CobW/COG0523 subgroup (referred to as “CobW” thereafter). A multiple sequence alignment of ZNG3 with Arabidopsis (*Arabidopsis thaliana*) ZNG1 and ZNG2, budding yeast (*Saccharomyces cerevisiae*) Zng1p (Pasquini et al., 2022; Weiss et al., 2022; Zhang et al., 2023), and *Chlamydomonas* proteins containing the CobW domain (Blaby‐Haas & Merchant, 2023) revealed the presence of a GCxCC motif required for metal binding between the Switch I and Walker B motifs of ZNG3, along with a good conservation of all other canonical motifs (Figure S2). This result suggests that ZNG3 may be implicated in the delivery of metal ions to client proteins via GTP hydrolysis (Haas et al., 2009; Jordan et al., 2019).

Some CobW proteins are predicted to be involved in Zn distribution due to the induction of their encoding genes under Zn deficiency (Coneyworth et al., 2012; Haas et al., 2009; Ogo et al., 2015). We therefore surveyed the expression of *ZNG3* and other genes encoding CobW domain‐containing proteins in *Chlamydomonas* using published RNA‐seq datasets probing the transcriptome as a function of Zn availability (Figure 2b left panel, Figure S3; Hong‐Hermesdorf et al., 2014; Malasarn et al., 2013). The Chlamydomonas genome encodes 11 proteins with a CobW domain, and another three that contain only the CobW C portion (Figure S3). Like *CIA5* mRNA abundance, *ZNG3* transcript levels were largely constant over a wide range of conditions (Figure 2b, left panel). Also similar to *CIA5* in the same dataset, *ZNG3* transcript abundance transiently rose upon Zn resupply to Zn‐deficient cultures (Figures 1e and 2b, left panel). Since *CIA5* was constitutively expressed under varying Zn supply (Figure 1e), while CIA5 was detected only under Zn‐replete conditions (Figure 1f), we checked ZNG3 abundance under Zn‐replete and Zn‐limited conditions in a previously published proteome study (Strenkert et al., 2023; Figure 2c). Similar to CIA5, ZNG3 protein abundance was about 14‐fold lower in the proteome of Zn‐limited cells than in Zn‐replete cells (Figure 2c). We also performed an immunoblot using ZNG3‐specific antibodies generated for this study, which confirmed the specific accumulation of ZNG3 under sufficient Zn supply (Figure 2d). While the presence of Zn was required for ZNG3 to accumulate, its interacting partner CIA5 was not, as ZNG3 was detected in both *cia5* mutants (Figure 2e). These observations suggest that CIA5 is not required for ZNG3 protein abundance.

Taken together, we identified ZNG3, a member of the metal‐binding CobW/COG0523 subgroup of the G3E family of P‐loop GTPases, as a CIA5 interactor. Unlike the other members *ZNG1* and *ZNG2* (Figure S3), *ZNG3* transcript abundance is not altered with respect to Zn nutrition, although Zn is required for ZNG3 accumulation.

### *ZNG3* is a target of CIA5 activation in low CO_2_ environments

We also examined the effects of carbon supply on the transcript abundance of *ZNG3* (Figure 2c, right panel) and other genes involved in Zn assimilation and distribution (Figure S3) using published RNA‐seq datasets. *ZNG3* transcript levels increasingly accumulated in wt *Chlamydomonas* cultures grown in LC, but not in the corresponding *cia5* mutant, in which *ZNG3* expression remained low (Figure 2b, right panel). This result indicates that *ZNG3* is a target of CIA5, inducing its expression during activation of the CCM. Among *Chlamydomonas* genes encoding CobW proteins, *ZNG3* expression was most similar to that of Cre16.g692901, as its transcript levels were not affected by Zn supply but were induced in a CIA5‐dependent manner under low CO_2_ conditions (Figure S3). *ZCP1* and *ZCP2* expression were solely induced in low Zn; *ZNG2* and Cre09.g801069 responded to low CO_2_ (CIA5‐dependently) and low Zn. Cre16.g658000 was the only CobW protein‐encoding gene repressed by Zn, while four other genes, *ZNG1*, Cre03.g175700, Cre12.g536900, and Cre14.g613200, were not affected by either treatment (Figure S3).

Of the three genes encoding CobW proteins without the GTPase domain (CobW C only), *LCI15* expression showed a pattern very similar to that of *ZNG3*. The expression of the two other CobW C genes was also induced in low CO_2_ in wt but not the *cia5* mutant, as well as in low Zn, unlike *ZNG3* (Figure S3). We also surveyed *CIA5* and *ZNG3* expression over the course of a diurnal cycle in phototrophically grown cultures synchronized by 12‐h dark/12‐h light cycles (Strenkert et al., 2019; Zones et al., 2015). *CIA5* and *ZNG3* transcript levels followed the same rhythmic pattern, with their mRNA abundance reaching their peaks directly after the onset of light (Figure 2f). The transcript levels of both genes anticipated dawn before declining sharply after (Figure 2f), suggesting that CIA5 is needed early in the day. Cre16.g692901 and *LCI15* diurnal profile again was very similar to that of *ZNG3* and *CIA5*, while other CobW genes showed various behaviors over the diurnal cycle (Figure S3).

### ZNG3 is a nuclear protein regardless of CO_2_ status

**Figure 3:**
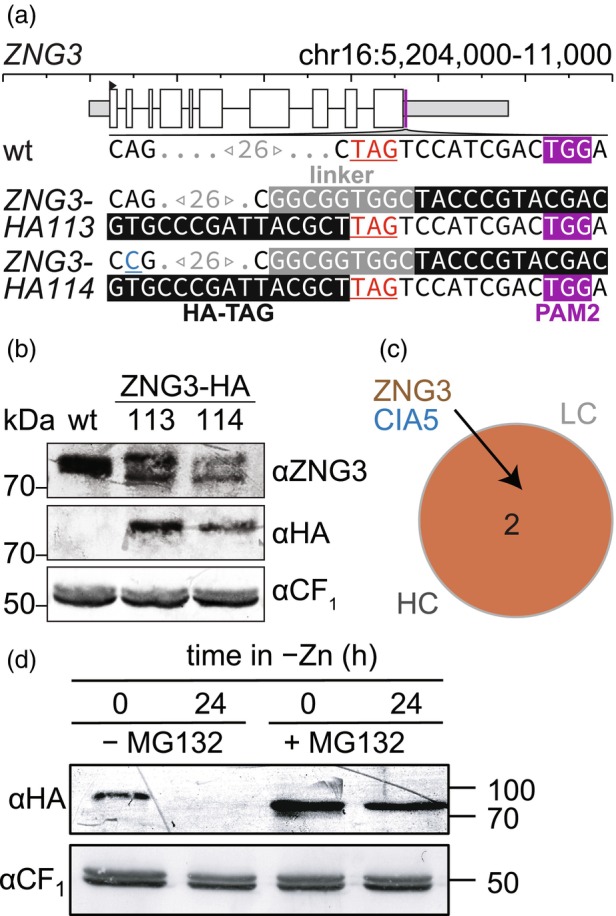
ZNG3 interacts with Cia5 independently of CO_2_ availability. (a) Diagram of the *ZNG3* locus showing the PAMs (purple) and target sites used to generate *ZNG3‐HA* knock‐in strains using Cas9. A short linker (gray) and the sequence encoding the HA epitope (black) were inserted in‐frame before the stop codon of *ZNG3*, yielding strains *ZNG3‐HA113* and *ZNG3‐HA114*; the two strains differ by one nucleotide, highlighted in blue font. (b) Immunoblot analysis of ZNG3 in wt and *ZNG3‐HA* strains. Total protein was extracted from cultures of wt and the two *ZNG3‐HA* strains grown under Zn‐replete conditions and analyzed as described in Figure 1d. Immunoblotting was performed with anti‐ZNG3 and anti‐HA antibodies. CF_1_ served as loading control. (c) Venn diagram showing the extent of overlap in proteins identified as ZNG3 interactors. Total protein from the two *ZNG3‐HA* strains grown phototrophically (5% CO_2_ [HC], or 0.04% CO_2_ [LC]) was subjected to immunoprecipitation with anti‐HA antibodies, followed by identification by LC–MS/MS. Only the number of identified proteins in both *ZNG3‐HA* strains but not in wt controls are shown. (d) ZNG3 is degraded by the proteasome under Zn‐deficient conditions. ZNG3‐HA113 was subjected to Zn‐replete (+) or Zn‐deficient (−) conditions alone or with the addition of the proteasome inhibitor MG132. CF1 served as loading control. Shown is one of two independent experiments.

To identify other potential ZNG3‐interacting proteins and to validate the ZNG3–CIA5 interaction, we introduced a sequence encoding a HA‐tag and a small, flexible glycine linker just upstream of the stop codon of the native *ZNG3* locus (Figure 3a). Here, we used a palindrome‐adjacent motif (PAM) compatible with Cas9 that was present in close proximity to the target site in *ZNG3*; several attempts to use a more distant PAM for CPf1 were unsuccessful. We obtained two independent *ZNG3‐HA* strains, named *ZNG3‐HA113* and *ZNG3‐HA114*, with no mutations or frameshifts that might alter ZNG3 translated from the edited locus (Figure 3a). We detected native ZNG3 in wt and the ZNG3‐HA fusion protein at an expected, larger molecular mass when using anti‐ZNG3 antibodies in the *ZNG3‐HA113* and *ZNG3‐HA114* strains (Figure 3b). We also observed a second, smaller band in the two edited strains when using anti‐ZNG3 antibodies, not seen in wt cells, potentially indicating the removal of the HA‐tag from a fraction of the protein. Since the full‐length protein was detected by the anti‐HA antibodies, we performed IPs on cell lysates from the two independent *ZNG3‐HA* strains grown under LC or HC conditions, followed by identification of coprecipitated protein by LC–MS/MS. Using the same criteria as for CIA5‐interacting proteins, we identified two proteins: the bait protein ZNG3 itself, as well as CIA5 (Figure 3c, Data S2). Based on our filtering criteria, we did not identify any other protein under LC or HC conditions. We took advantage of the *ZNG3‐HA* strains to test whether the absence of ZNG3 accumulation in Zn‐deficient cells might reflect degradation via the UPS; indeed, treatment with MG‐132 resulted in the accumulation of ZNG3‐HA in Zn‐deficient cells, indicating that ZNG3‐HA, and likely native ZNG3, is targeted for degradation via the UPS under Zn deficiency (Figure 3d).

**Figure 4:**
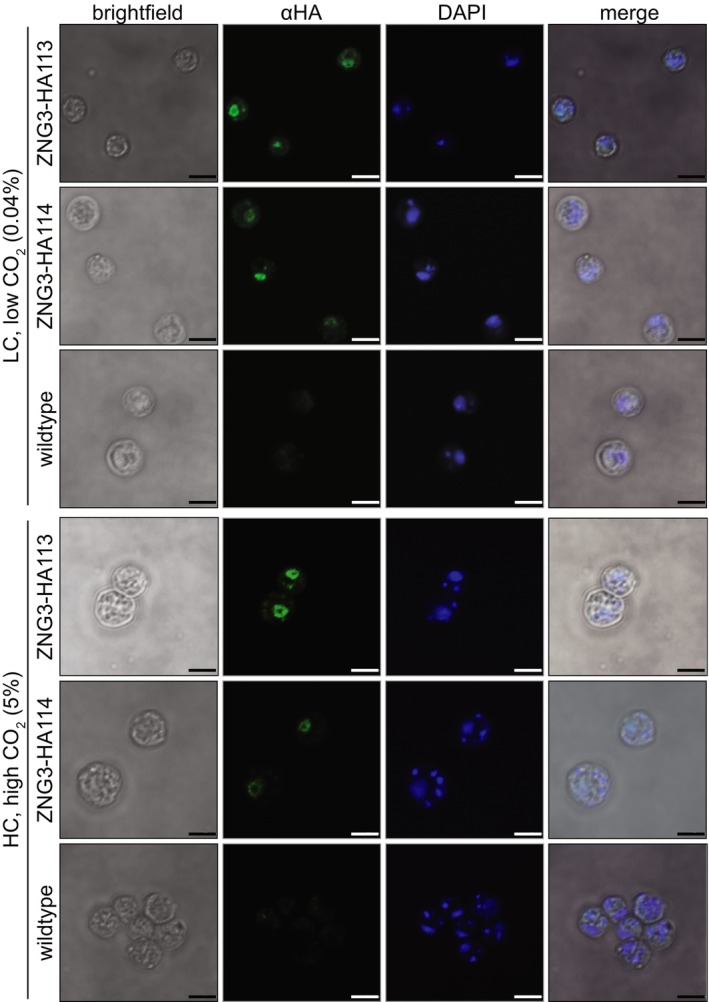
ZNG3 localizes in the nucleus independently of CO_2_ availability. Representative confocal micrographs showing the localization of ZNG3‐HA as determined by immunofluorescence with anti‐HA antibodies and a secondary antibody conjugated to Alexa Fluor 488. Cells from wt, *ZNG3‐HA113*, and *ZNG3‐HA114* were grown under LC or HC conditions before sample collection. Nuclei were stained with the fluorescent dye 4′,6‐diamidino‐2‐phenylindole (DAPI) included in the mounting solution. Scale bars, 5 μm.

We investigated the subcellular localization of ZNG3‐HA in the *ZNG3‐HA* strains grown phototrophically in LC or HC conditions via immunofluorescence with anti‐HA antibodies, using wt cells as negative control (Uniacke et al., 2011). Nuclei were stained with the fluorescent dye 4′,6‐diamidino‐2‐phenylindole (DAPI). We detected fluorescent signals for ZNG3‐HA in the nucleus of cells grown under HC and LC conditions, with complete overlap with the DAPI signal (Figure 4), similar to that previously observed for CIA5 (Wang et al., 2005). Notably, immunostaining for ZNG3‐HA revealed a ring‐like pattern within the nucleus, in contrast to the even staining seen with DAPI throughout the nucleus. DAPI also stains polyphosphates (polyP), a major component of acidocalcisomes. Importantly, ZNG3‐HA localized only to the nucleus of cells grown under HC conditions, and not to acidocalcisomes (Figure 4).

In summary, we experimentally validated the predicted nuclear localization of ZNG3, which is not affected by CO_2_ supply. This result is in agreement with the constitutive interaction of ZNG3 with Cia5, as Cia5 is also a nuclear protein.

### ZNG3 is important for growth when CO_2_ is not limiting

**Figure 5:**
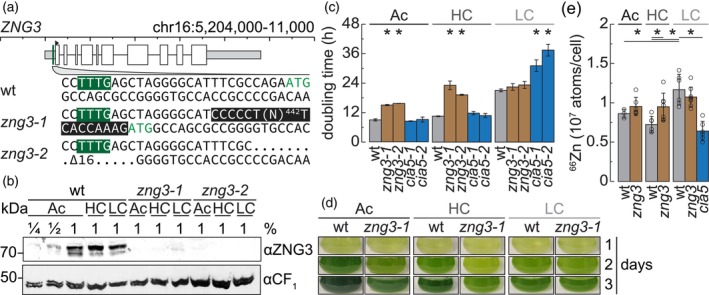
*zng3* mutants grow poorly under high CO_2_ conditions. (a) Diagram of the *ZNG3* locus showing the PAMs (green) upstream of the start codon used to generate mutants. The introduced gene edits in *zng3‐1* and *zng3‐2* are shown below. Insertion of part of the *THI4* riboswitch DNA fragment in *zng3‐1* is shown in black. (b) Immunoblot analysis of ZNG3 abundance in wt, *zng3‐1*, and *zng3‐2* mutants grown photoheterotrophically (with acetate as sole carbon source, Ac), or phototrophically via bubbling with 5% CO_2_ (HC) or 0.04% CO_2_ (LC). Immunoblotting was performed with anti‐ZNG3 antibodies. CF_1_ served as loading control. (c) Doubling time of wt, *zng3‐1*, *zng3‐2*, *cia5‐1*, and *cia5‐2* cultures grown as in (b). Cell counts were obtained at 24‐h intervals. Doubling times were calculated from data obtained during exponential growth. Data are means ± SD from three independently grown cultures. Asterisks indicate significant differences (two‐sided *t*‐test, *P* < 0.05). (d) Representative photographs of culture flasks taken on days 1, 2, and 3 after inoculation. The cultures were grown as in (b). (e) Zn content of wt and *zng3* mutants grown photoautotrophically under HC or LC conditions, or photoheterotrophically (Ac), determined by ICP‐MS/MS and normalized to cell number. Data are means ± SD from 6 to 9 independent experiments; individual data points are shown as open circles.

To determine the involvement of the metal GTPase ZNG3 during the establishment of the CCM and in Zn homeostasis, we generated independent *zng3* mutants using CRISPR‐mediated gene editing. Our initial attempts to introduce in‐frame stop codons in the *ZNG3* locus were not successful, suggesting that ZNG3 might be an essential gene. We therefore turned to a thiamine‐repressible system by introducing a *THIAMINE 4* (*THI4*) riboswitch upstream of the *ZNG3* start codon (Schmollinger et al., 2026). Among transformants, we obtained two independent mutants, designated *zng3‐1* and *zng3‐2* (Figure 5a). *zng3‐1* harbors an insertion of a truncated, non‐functional 442‐bp *THI4* riboswitch fragment within the 5′ untranslated region (5′ UTR) of *ZNG3*, containing several open reading frames (ORFs) in all reading frames, while *zng3‐2* carries a 16‐bp deletion that included the *ZNG3* start codon (Figure 5a). In immunoblots on total cell lysates of wt, *zng3‐1*, and *zng3‐2* mutants using anti‐ZNG3 antibodies, *zng3* mutants appeared to accumulate little to any ZNG3 (Figure 5b). Like CIA5, ZNG3 protein abundance was comparable across cells grown under LC or HC conditions or in the presence of acetate (Figure 5b).

To test the effect of loss of ZNG3 function on the ability of cells to grow in different carbon regimes, we grew wt and the *zng3* mutants photoheterotrophically or phototrophically under HC and LC conditions (Figure 5c,d). For comparison, we included the two independent *cia5* mutants. We hypothesized that the potential Zn delivery by ZNG3 to CIA5 would be required for successful CCM establishment and therefore expected *zng3* mutants to grow poorly under low CO_2_ conditions, similar to the published phenotype of *cia5* mutants (Moroney et al., 1989). We recapitulated this expected growth phenotype with our *cia5* mutants, which were indeed asymptomatic when grown photoheterotrophically or phototrophically under HC conditions, but had slower growth rates under LC conditions, as evidenced by their longer doubling times (Figure 5c). Surprisingly, LC‐grown *zng3* mutants exhibited growth rates comparable to that of wt, with an average doubling time of 22 h compared with 21 h for wt cells. However, the two *zng3* mutants did exhibit a significant growth defect when grown phototrophically under HC conditions or in the presence of acetate as sole carbon source (Figure 5c,d). We conclude that ZNG3 is not required for successful establishment of the CCM but does contribute to growth under HC and photoheterotrophic conditions, where loss of CIA5 does not result in a growth phenotype. We also measured the Zn content of the *zng3* mutants in all growth regimes to assess their ability to adjust their Zn quota in response to low CO_2_ availability. Unlike the wt, the Zn content of *zng3* cells was not significantly different between HC and LC, possibly due to the higher intracellular Zn levels in *zng3* mutants relative to wt under HC conditions (Figure 5e).

### The expression of CCM genes is induced in wt and *zng3* mutants under low CO_2_ conditions

**Figure 6:**
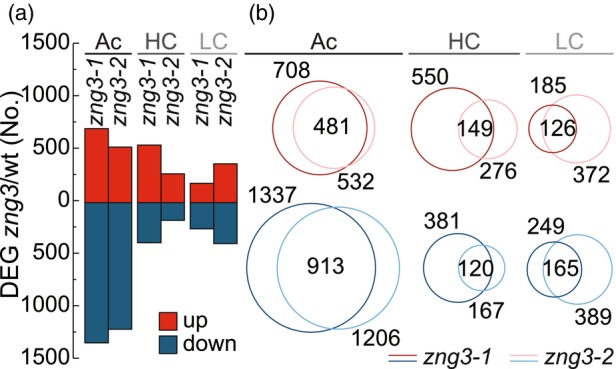
Identification of differentially expressed genes between wild‐type and *zng3* mutants. Cultures of wt, *zng3‐1*, and *zng3‐2* mutants were grown photoheterotrophically (with acetate, Ac), or phototrophically with bubbling with 5% CO_2_ (HC) or 0.04% CO_2_ (LC). RNA‐seq was performed on all samples, and differentially expressed genes were identified with the criteria absolute log_2_(fold‐change) >1 and adjusted *P*‐value <0.05 with Cuffdiff (v2.2.1) (Trapnell et al., 2012). (a) Number of differentially expressed genes (DEGs) between *zng3‐1* or *zng3‐2* and wt under the indicated conditions. (b) Proportional Venn diagrams showing the number of significant upregulated (red) or downregulated (blue) DEGs in each pairwise comparison and their overlap.

**Figure 7:**
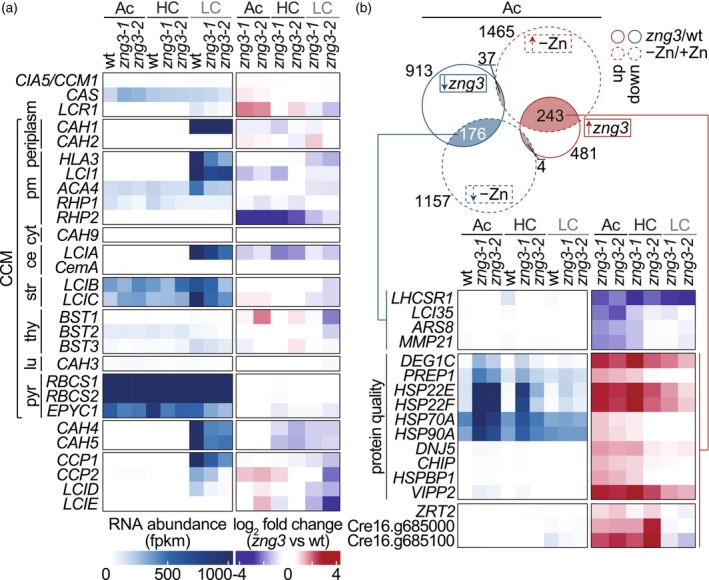
The expression of genes encoding proteins involved in plastid quality control and Zn metabolism is induced in *zng3* mutants. (a) Heatmap representation of transcript abundance in FPKM (left) or log_2_(fold‐change) values between *zgn3‐1* or *zgn3‐2* and wt (right), based on RNA‐seq data, for genes encoding proteins involved in the CCM. Wt, *zgn3‐1*, and *zgn3‐2* cultures were photoheterotrophically (acetate, Ac) or phototrophically via bubbling with 5% CO_2_ (HC) or air (LC). Data are means from six wt cultures (two independent strains, three replicates each) and from three independent cultures of each mutant. (b) Venn diagram showing the extent of overlap for DEGs identified by RNA‐seq between Zn‐deficient and Zn‐replete cultures (from Hong‐Hermesdorf et al., 2014) and between *zng3* mutants and wt cultures (this study). Heatmaps and samples are as described in (a). The genes shown in the heatmap exhibited a consistent, significant response in both *zng3* mutants in at least one growth mode. A more expansive list of genes involved in these pathways, including those not changing in *zng3* mutants, can be found in Figures 4, 5, 6.

To explore the consequences of the loss of ZNG3 on Zn and carbon metabolism in more detail, we performed RNA‐seq on wt cells and the two independent *zng3* mutant strains grown phototrophically in LC, HC, or photoheterotrophically with acetate. We identified differentially expressed genes (DEGs) between wt and each *zng3* mutant strain for each growth condition, resulting in 1394 (481 upregulated, 913 downregulated) DEGs for photoheterotrophic cells, 269 (149 up, 120 down) DEGs for HC‐grown cells, and 291 (126 up, 165 down) DEGs for LC‐grown cells (Figure 6a,b). We captured the expected transcriptomic signature of cells under LC and HC conditions, including the characteristic induction of structural CCM genes encoding the periplasmic carbonic anhydrase *CAH1*, the HCO_3_^−^/CO_2_ importers HIGH LIGHT‐ACTIVATED 3 (*HLA3*) and *LCI1*, the chloroplast bicarbonate importer LCIA (Förster et al., 2023), and the mitochondrial carbonic anhydrases CAH4 and CAH5 (Figure 7a, Figure S4, Data S3). These known *LCI* genes were still induced in *zng3* mutants, although not as strongly induced as in wt (Figure 7a), for example *HLA3* (Gao et al., 2015), the outward proton pump gene *AUTOINHIBITED Ca*2^+^‐*ATPASE 4* (*ACA4*) (Mackinder et al., 2017), *LCIC* (Yamano et al., 2010), *CAH4* and *CAH5*, as well as *ESSENTIAL PYRENOID COMPONENT 1* (*EPYC1*), which is involved in pyrenoid organization (Atkinson et al., 2019; Mackinder et al., 2016; Figure 7a). The expression of most of these genes is dependent on CIA5 (Fang et al., 2012; Rai et al., 2021), suggesting that CIA5 is largely functional in *zng3* mutants, consistent with *zng3* mutants being asymptomatic when grown under LC conditions (Figure 5d). The expression levels of almost all genes involved in the CCM were moderately to strongly induced in wt and the *zng3* mutants grown under LC conditions, while higher carbon supply (HC or Ac) resulted in higher expression of some CCM‐related genes in the *zng3* mutants than in wt, including *LOW‐CO*_*2*_ *STRESS RESPONSE 1* (*LCR1*), *LCIC*, *BESTROPHIN‐LIKE 1* (*BST1*), and *CCP2*, although far below the induction seen under LC conditions (Figure 7a).

Taken together, loss of ZNG3 function minimally affects the expression of genes involved in the CCM, only slightly dampening the extent of induction under LC conditions, in agreement with the lack of phenotype under this growth condition.

### Zn deficiency genes are derepressed in *zng3* mutants grown with high carbon supply

Since ZNG3 is a potential Zn metallochaperone, we wondered if the expression of genes encoding proteins involved in Zn homeostasis would be affected in the *zng3* mutants. Indeed, we observed a specific upregulation in the expression of two genes encoding CobW C proteins (Cre16.g685000 and Cre16.g685100, Figure S1) under HC conditions and in the presence of acetate in the *zng3* mutants compared with wt (Figure 7a). These genes were previously shown to be induced under Zn deficiency (Hong‐Hermesdorf et al., 2014), and are not expressed in a *cia5* mutant grown under LC conditions (Figure S3). While *ZRT2* expression levels were higher in the *zng3* mutants than in wt grown with ample carbon supply (HC and Ac), they were far lower than in Zn‐limited cells (Hong‐Hermesdorf et al., 2014) (Figure 7a, Figure S3). Expression of other genes encoding proteins involved in Zn import and distribution, including ZRT1, ZRT3, ZINC‐RESPONSIVE COG0523 DOMAIN‐CONTAINING PROTEIN 2 (ZCP2), ZNG2, and the CobW domain‐containing proteins encoded by Cre12.g536900 and Cre16.g658000, was also higher in the presence of acetate and under HC conditions (Figure 7b, Figure S5). A comparison of DEGs in *zng3* relative to wt and those previously identified in response to Zn limitation (Hong‐Hermesdorf et al., 2014) revealed that a substantial fraction of shared DEGs between Zn‐limited wt cells and the Zn‐replete *zng3* mutants (Figure 7b).

Together, these results suggest that ZNG3 contributes to the repression of genes encoding components of Zn metabolism (ZRT transporters, ZCP2, other CobW proteins), with global expression adjustments in large part being similar to metabolic adjustments observed under Zn deficiency.

### The (plastid) protein quality control system is induced in *zng3* mutants

Genes encoding chaperones, such as the chloroplast small heat shock proteins (sHSPs) HSP22E and HSP22F, and other proteins involved in protein quality control were among the most highly induced in *zng3* mutants (Figure 7b, Figure S6). The amplitude of *HSP22E* and *HSP22F* induction was greater under conditions in which *zng3* mutants showed a significant growth defect (Figure 4a, Figure S6). The chaperone activity of HSP22E and HSP22F maintains plastid protein homeostasis during heat stress in *Chlamydomonas*, although clients of both sHSPs also include proteins involved in starch biosynthesis and CO_2_ assimilation (Rütgers et al., 2017). The higher *HSP22E* and *HSP22F* transcript levels raised the possibility that *zng3* mutants accumulate unfolded proteins within their chloroplasts. In support of this hypothesis, the expression of a gene encoding the chloroplast‐localized protease DEGRADATION OF PERIPLASMIC PROTEINS 1 (DEG1C) (Theis et al., 2019) was also upregulated in *zng3* mutants, with a greater induction in cells grown in the presence of acetate or under HC conditions (Figure 7a). Interestingly, it was previously shown that a small subset of proteins involved in establishing the CCM accumulate in *deg1c* mutants, like plastid LCIB, LCIC, and LHCSR1, indicating their dependence on DEG1C for quality control (Theis et al., 2019). Transcript levels of *LCIC* (only under LC conditions) and *LHCSR1* (all conditions) were lower in *zng3* mutants relative to wt.

In summary, RNA‐seq analysis suggests that components of the CCM are generally not largely misregulated in *zng3* mutants under LC conditions, indicating that ZNG3 is not required to establish the CCM in *Chlamydomonas*. Instead, a fraction of genes induced in *zng3* mutants also respond to Zn supply, suggesting that ZNG3 may be involved in coordinating the response to low Zn environments. Most prominently, the expression of two genes encoding metallochaperones with a CobW C domain was highly induced in *zng3* mutants. These two genes are also possible targets of CIA5 in low CO_2_ environments. Genes encoding chloroplast‐targeted sHSPs and DEG1C with possible roles in the CCM were also highly induced in *zng3* mutants grown in the presence of high CO_2_ supply. Taken together, the physical connection between ZNG3 and CIA5 may offer a platform to coordinate Zn and CO_2_ assimilation.

### CIA5 is conditionally epistatic to ZNG3

**Figure 8:**
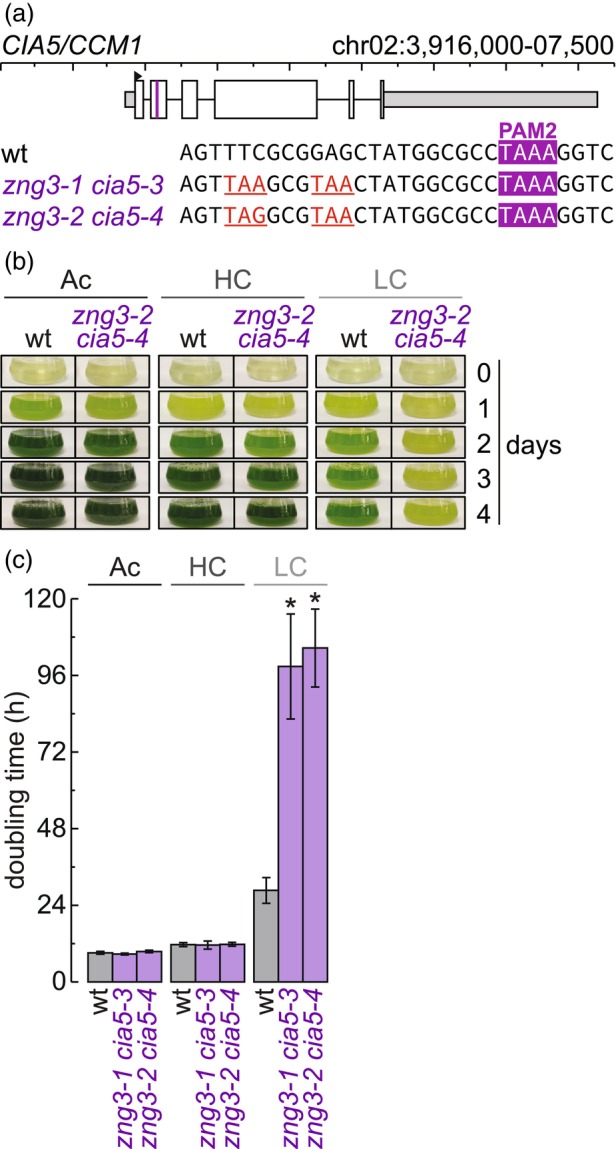
*zng3 cia5 double* mutants show compromised growth under low CO_2_ conditions. (a) Diagram of the *Cia5* locus as illustrated in Figure 1 showing the PAMs (magenta) used to generate mutants in the *zng3‐1* and *zng3‐2* backgrounds by introducing early stop codons. The edits introduced in *Cia5* in *zng3‐1 cia5‐3* and *zng3‐2 cia5‐4* are shown below. (b) Representative photographs of culture flasks of wt, *zng3‐1 cia5‐3*, and *zng3‐2 cia5‐4* double mutants taken on days 0, 1, 2, 3, and 4 after inoculation. The cultures were grown photoheterotrophically (with acetate, Ac), or phototrophically via bubbling with 5% CO_2_ (HC) or 0.04% CO_2_ (LC). (c) Doubling time of wt, *zng3‐1 cia5‐3*, and *zng3‐2 cia5‐4* double mutants grown as in (b). Cell counts were obtained at 24‐h intervals. Doubling times were calculated from data obtained during exponential growth. Data are means ± SD from three independently grown cultures. Asterisks indicate significant differences (two‐sided *t*‐test, *P* < 0.05).

We generated *zng3 cia5* double mutants by inserting in‐frame stop codons into the first exon of the *CIA5* locus in the *zng3‐1* and *zng3‐2* mutant backgrounds, using the same single‐guide RNA (sgRNA) used for the *cia5* single mutants (Figure 8). We grew the two *zng3‐1 cia5‐3* and *zng3‐2 cia5‐4* double mutants and the wt strain photoheterotrophically or phototrophically (HC or LC) to determine their doubling times (Figure 8). The *zng3‐1 cia5‐3* and *zng3‐2 cia5‐4* double mutants grew like wt under HC and Ac conditions, with normal doubling times, suggesting that CIA5 is epistatic to ZNG3, acting downstream of ZNG3 with excess carbon supply. Notably, the double mutants grew very poorly under LC conditions, showing a more severe growth defect than that of the corresponding *cia5* single mutants (Figures 5 and 8), suggesting that CIA5 and ZNG3 provide independent contributions to fitness under LC conditions, in addition to specific roles under high carbon conditions.

## DISCUSSION

### Acclimation to low CO_2_ requires adjustments to Zn metabolism

Organisms have evolved sophisticated systems for Zn uptake, intracellular distribution, compartmentalization, and efflux to achieve cellular Zn homeostasis (Eide, 2006). Many of the components responsible for Zn transport and sequestration have been identified in phototrophs, although the underlying regulatory mechanisms remain largely unknown (Eide, 2006; Haas et al., 2009). Importantly, Zn is required for adequate chloroplast metabolism, photosynthesis, and CO_2_ assimilation. CobW/COG0523 domain‐containing metallochaperones are a group of evolutionary conserved proteins across prokaryotes and eukaryotes that deliver metals to individual target proteins, some functioning as Zn chaperones (Crouzet et al., 1991; Haas et al., 2009; Weiss et al., 2022). These proteins are premier candidates to facilitate Zn supply to phototroph‐specific targets in chloroplasts; in agreement with this idea, Arabidopsis mutants in the Zn metallochaperone gene *ZNG1* showed growth defects under low Zn supply and pronounced misregulation of genes encoding proteins involved in photosynthesis and carbon fixation (Zhang et al., 2023). The exact functions of CobW proteins and their direct clients remain, however, limited.

In this work, we dissected the connections between Zn supply, the CCM, and the transcriptional regulator CIA5 in *Chlamydomonas*, identifying the nucleus‐located protein ZNG3 as an interacting metal‐binding GTPase. Our finding thus links a critical regulator of the CCM to metal homeostasis in this green alga. Unlike other genes encoding Zn‐related CobW proteins (Haas et al., 2009), *ZNG3* expression levels did not change in response to Zn supply but increased following transfer from high CO_2_ to low CO_2_ (Figure 2c). We observed a similar expression pattern for a second, uncharacterized CobW gene (Cre16.g692901), and for *LCI15* encoding a CobW C domain‐containing metallochaperone; this induction under low CO_2_ conditions was dependent on CIA5 (Figure S3). The expression of most CobW genes was induced under low Zn and low CO_2_, as was that of high‐affinity Zn transporter genes from the *Zrt‐ and Irt‐related protein* (*ZIP*) family (*ZRT1–3*, Figure S3).

In all cases, the observed changes in transcript abundance depended on CIA5, indicating that phototrophic, low‐CO_2_ growth conditions impose a burden on cells to maintain Zn homeostasis. This observation is not surprising, as CAHs are highly abundant Zn‐containing proteins central to CCM function. Indeed, the expression levels of *CAH* genes, putative direct CIA5 targets, are markedly higher in low CO_2_ environments, which may be sufficient to explain the greater intracellular Zn quota of cells grown under these conditions (Figure 1c). In line with this idea, *Chlamydomonas* cells grown under low CO_2_ and Zn limitation exhibit a severe growth defect (Malasarn et al., 2013). Zn must therefore be delivered to most cellular compartments to support CCM function by providing the necessary cofactors to proteins that accumulate under low CO_2_, most prominently periplasmic CAH1, mitochondrial CAH4 and CAH5, and chloroplast LCIB–LCIC and CAH3 (Eriksson et al., 1996; Van & Spalding, 1999; Yamano et al., 2010). The concomitant induction of metallochaperone‐encoding genes under low CO_2_ conditions would facilitate Zn distribution to individual clients or cellular compartments. That these genes are under the transcriptional control of a regulator of the CCM would represent a simple yet elegant way to ensure functionality of the newly synthesized Zn‐containing proteins.

### Potential roles for the COBW domain‐containing metal GTPase ZNG3

One possible explanation for the growth defects observed in *zng3* mutants when grown under HC conditions and in the presence of acetate is that ZNG3 is a negative regulator of CIA5 under these conditions, which would lead to the de‐repression of CCM genes in the mutants and the establishment of a CCM, thus placing a significant metabolic demand on *zng3* mutants. However, we and another group did not see widespread de‐repression of *LCI* genes reported as putative direct CIA5 targets in *zng3* mutants (Figure 7; Shimamura et al., 2026). Indeed, while the two *LCI* genes *CAH1* and *LCIB* were slightly upregulated in *zng3* mutants, as were their encoded proteins (Shimamura et al., 2026), dozens of *LCI* genes were moderately downregulated, while expression of the remaining *LCI* genes remained unchanged. In agreement with the lack of a widespread, molecular phenotype in *zng3* mutants, LCIB was recently shown to display a typical pyrenoid localization in *zng3* mutants grown under very‐low CO_2_ conditions, indicative of CCM establishment, but showed a diffuse pattern throughout the cell under HC conditions (Shimamura et al., 2026).

Outside of acclimation to low CO_2_ conditions, we determined that ZNG3 regulates the expression of genes encoding proteins involved in metal distribution. Two such highly induced genes in *zng3* mutants encode proteins that contain a CobW domain like ZNG3 but are predicted to localize to different compartments (Figure 7b). In wt cells, both genes are induced in response to low Zn or CO_2_ supply and require CIA5 function for their higher expression under LC conditions (Figure S3), while they are derepressed under Zn‐replete, carbon‐rich growth conditions (Ac and HC). This observation suggests that the ZNG3–CIA5 complex adjusts Zn homeostasis outside of LC conditions. When *Chlamydomonas* cells are grown under carbon‐rich conditions and do not need a CCM, the CO_2_ status may be relayed via CIA5 to prevent Zn from being routed to the targets of these metallochaperones, via ZNG3. In the absence of ZNG3 function, Zn accumulated to higher levels than in wt cells when experiencing high CO_2_ supply (Figure 5). Thus, the above hypothesis is consistent with the observation that the expression of genes involved in plastid protein quality control (*HSP22E*, *HSP22F*, *DEG1C*) was upregulated in *zng3* mutants, under conditions where the CCM is not utilized (Figure 7b). The upregulation of these quality control genes could be a consequence of Zn ions being mistakenly trafficked to the plastid in *zng3* mutants when plastid Zn demand is low, possibly resulting in mis‐metalation of non‐Zn metal‐binding proteins, and causing protein misfolding (Hansen & Hilgenfeld, 2013; Haslbeck & Vierling, 2015). Alternatively, ZNG3–CIA5 may control the expression of *HSP22E*, *HSP22F*, and *DEG1C*, which on their own directly modulate the CCM. Among the targets of HSP22E and HSP22F in *Chlamydomonas* were four proteins involved in starch biosynthesis, including rbcL and RuBisCO activase (RCA) (Rütgers et al., 2017). *deg1c* mutants showed increased accumulation of the CCM‐related proteins LCIB, LCIC, LCI11, CCP1, LHCSR1, and proteins involved in starch biosynthesis and degradation, indicating a direct role for DEG1C in controlling the abundance and integrity of these CCM proteins (Theis et al., 2019). Notably, LHCSR1 is a target of both sHSPs and DEG1C. *LHCSR1* expression levels were lower under all growth conditions in the *zng3* mutants relative to wt, which might be a direct consequence of increased DEG1C production (Figure 7b). *VESICLE‐INDUCING PROTEIN IN PLASTIDS 2* (*VIPP2*) was also more highly transcribed in *zng3* mutants; VIPP2 was shown to be induced upon oxidative stress and under increased light exposure. Together with HSP22E, HSP22F, and DEG1C, VIPP2 might be involved in preventing or clearing damaged proteins from thylakoid membranes (Theis et al., 2020). It remains to be determined whether the upregulation of *LCIB* and *CAH1* in wt and *zng3* mutants represents a metabolic burden or if the higher Zn quota under low CO_2_ conditions results in significant reactive oxygen species (ROS) production. Either scenario or a combination of both could help explain the observed fitness penalty of HC‐grown *zng3* mutants.

### Connection between CIA5 and ZNG3

Based on previous work on the vertebrate and yeast ZNG1, the mode of action for metal‐containing GTPase of the CobW family is proposed to involve GTP hydrolysis driving Zn transfer from the metallochaperone to the catalytic site of its client (Pasquini et al., 2022; Weiss et al., 2022). The client protein for vertebrate ZNG1s was demonstrated to be the Zn metalloprotease Methionine aminopeptidase 1 (METAP1), whose activity is maintained by ZNG1 when cellular Zn levels are low (Weiss et al., 2022). We did not see differential association between CIA5 and ZNG3 when cells were grown under different carbon supply, suggesting that ZNG3 does not simply metalate CIA5 in response to CO_2_ status, and therefore cannot easily explain the activation of CIA5 and the CCM activation under LC conditions. Instead, we hypothesize that CIA5 metalation is maintained based on the presence of Zn, since both ZNG3 and CIA5 have metal‐binding domains, and both proteins were degraded via the UPS in cells grown in Zn‐limited growth medium, although their encoding genes are constitutively expressed (Figures 1e,f and 2c–e). An earlier study on mutant variants of CIA5 with mutations in the Zn‐binding cysteines in the first C_2_H_2_ Zn finger led to loss of CIA5 function and its rapid degradation (Kohinata et al., 2008). ZNG3 abundance is unchanged in *cia5* mutants (Figure 2) and the observation in another recent study (Shimamura et al., 2026) in which CIA5 accumulation was unaffected in *zng3* mutants, suggests that the CIA5–ZNG3 interaction is not crucial for maintaining their respective protein abundance.

In summary, low Zn supply removes a key regulator required to establish the CCM in *Chlamydomonas*, CIA5, together with its constitutive interaction partner, ZNG3. The active degradation of these two proteins under low Zn supply blocks the establishment of the CCM, which would entail the production of abundant Zn‐containing proteins in the absence of sufficient Zn. The *zng3* mutants do not phenocopy *cia5* mutants, grow like wt under low CO_2_ conditions, and still induce the expression of genes encoding central CCM components. We propose that the metal‐containing proteins ZNG3 and CIA5 form a complex that may represent a nucleus‐localized regulatory gateway connecting Zn and carbon metabolism.

## MATERIAL AND METHODS

### Chlamydomonas growth conditions

The *Chlamydomonas* parental strain used for all experiments was CC‐425. Cells were grown in Tris‐acetate phosphate (TAP) medium or high salt medium from Sueka (HSM) prepared with revised trace elements (Kropat et al., 2011) with constant agitation at 120 rpm in a shaking incubator (Multitron, Infros HT, Annapolis Junction, MD, USA) at 24°C in continuous light (60 μmol m^−2^ s^−1^) (Kropat et al., 2011). Phototrophically grown cultures were bubbled with humidified air and CO_2_ mixtures (5% [v/v] CO_2_ [high CO_2_, HC] or 0.04% [v/v] CO_2_ [low CO_2_, LC]) as indicated.

For proteasome inhibitor experiments, strains were grown in TAP medium until they reached early, mid‐exponential growth phase. Cells were collected by centrifugation at 3500 ***g*** and 24°C for 3 min. Supernatant was removed and cells were washed with 1 mM EDTA to remove cell surface bound metals. Cells were collected by centrifugation at 3500 ***g*** and 24°C for 3 min and resuspended in TAP medium without Zn supplementation containing either 0.1% DMSO or 20 μM MG‐132 in 0.1% DMSO as indicated.

### Generation of Chlamydomonas mutant strains via gene editing with Cpf1 or Cas9

*Chlamydomonas* CC‐425, an arginine auxotrophic strain with a thin cell wall, was used as the recipient strain for transformation. Briefly, cells were mixed with the plasmid pMS666 (digested with HindIII) or pHR11 (digested with EcoRI) carrying the *ARG7* gene and a ribonucleoprotein (RNP) complex consisting of a single‐guide RNA (sgRNA) targeting the protospacer‐adjacent motif (PAM) TTTV for *Lachnospiraceae bacterium* Cpf1, as described in Ferenczi et al. (2017), Pham et al. (2022) and Strenkert et al. (2024), or NGG for *Streptococcus pyogenes* Cas9. ssODN templates with the intended edits were added to the mixture prior to electroporation. The wt control strains used in this study were generated by transforming CC‐425 with plasmid pHR11 (digested with EcoRI) carrying the ARG7 gene to restore arginine prototrophy. The sequences of the PAMs and sgRNAs are listed in Table S1. Primary transformants were selected on arginine‐free TAP agar (1.5% [w/v]) plates and screened by colony PCR with the primers listed in Table S1. For this purpose, *Chlamydomonas* cells were scraped from the surface of the TAP agar plates and resuspended in 100 μL of TE buffer (10 mM Tris–HCl, 1 mM EDTA, pH 8.0). The cell suspension was then heated at 95°C for 10 min, followed by vigorous vortexing and centrifugation at 500 ***g*** for 5 min at 24°C. The supernatant was used as template for genotyping PCR using either GoTaq Green Mastermix (Promega, Medison, WI, USA) with the addition of 1 M betaine or for quantitative PCR (qPCR) with SYBR Green Mastermix (Bio‐Rad) and addition of 1 M betaine. Screening for successful integration was conducted using diagnostic primers that specifically amplify either transgene‐derived or endogenous DNA sequences at the integration site. For screening of *Cia5‐HA* strains, individual colonies were grown on TAP medium and total cell lysates were separated by SDS‐PAGE, transferred to nitrocellulose membranes, and probed with anti‐HA antibody as described below.

After selecting candidates (mutant strains or strains producing tagged proteins), DNA sequences were assessed by PCR amplification of the gene‐edited region with sequencing primers (Table S1) and GoTaq Green Mastermix (Promega, Medison, WI, USA) with the addition of 1 M betaine. The PCR products were separated on a 1% (w/v) agarose gel for electrophoresis in 1× Tris‐acetate EDTA (TAE) buffer. The DNA fragments were then extracted using an E.Z.N.A. Gel Extraction Kit (Omega Bio‐tek, Norcross, GE, USA) according to the manufacturer's instructions and subjected to Sanger sequencing.

### Immunofluorescence

Immunofluorescence was performed according to a published protocol (Uniacke et al., 2011). In brief, after coating glass coverslips with poly D‐lysine (A38904, Life Technologies), cells were allowed to adhere for 4 min before excess cells were washed three times with sterile 1× phosphate‐buffered saline (PBS). Coverslips were then placed in methanol pre‐cooled to −20°C for 10 min. Samples were air dried for at least 30 min and rehydrated in sterile 1× PBS for 20 min before blocking for 1 h at room temperature in blocking solution (0.5% [w/v] BSA in 1× PBS). Primary anti‐HA antibodies (H6908, Sigma Aldrich, St. Louis, MO, USA) were used at a dilution of 1:1000 (v/v). The secondary antibody was Alexa Fluor 488‐conjugated goat anti‐rabbit antibody (A32731, Thermo Fisher, Waltham, MA, USA) and was used at a 1:1000 (v/v) dilution. After immunoblotting, coverslips prepared as above were mounted onto clean glass slides using Antifade mounting medium containing DAPI (D1306, Thermo Fisher). Micrographs were captured on a Nikon A1Rsi Laser Scanning Confocal Microscope.

### Quantitative elemental analysis

A volume of cell culture corresponding to 5 × 10^7^ cells was collected during exponential growth at a culture density of 3–8 × 10^6^ cells/mL by centrifugation at 1424 *g* for 3 min in a 50‐mL Falcon tube. The cell pellets were washed once in 50 mL 1 mM Na_2_EDTA pH 8.0 to remove cell surface‐associated metals, and once in Milli‐Q water. The cell pellets were stored at −20°C until processing, which entailed overlaying the pellets with 286 μL 70% (v/v) nitric acid, followed by digestion at 65°C overnight. The digested cells were then diluted to a final nitric acid concentration of 2% (v/v) with Milli‐Q water. The contents of metal ions, sulfur, and phosphorus were determined by inductively coupled plasma–tandem mass spectrometry (ICP‐MS/MS) on an Agilent 8800 Triple Quadropole ICP‐MS instrument against a calibration standard (Agilent 5183‐4688). ^45^Sc served as an internal standard (Inorganic Ventures MSY‐100PPM). The levels of ^66^Zn were determined in MS mode directly using helium (He) in a collision reaction cell. An average of three technical replicate measurements was used for each individual biological sample. The variation between technical replicate measurements never exceeded 5% for any individual sample. Four technical replicates from three to nine samples from independent cultures per condition were used to determine the average abundance and variation between cultures shown in all figures; individual points indicate the abundance of each individual independent culture.

### Antibody production and immunodetections

Antibodies targeting ZNG3 were produced by Labcorp via immunization of rabbits using the subcutaneous implant procedure of rabbits following a 118‐day protocol with the synthetic peptide Ac‐ERNPKRRAKRLHDLC‐NH_2_. The antibodies against ZNG3 were affinity‐purified by Labcorp using the immobilized peptide. For immunoblot analysis, 2 × 10^7^ cells were collected from cultures at mid‐logarithmic phase by centrifugation at 3000 ***g*** for 3 min at 4°C. For protein quantification, the cell pellets were resuspended in 50–200 μL 10 mM sodium phosphate buffer pH 7.0 (4.23 mM NaH_2_PO_4_, 5.77 mM Na_2_HPO_4_) with or without protease inhibitor cocktail (Roche) and stored at −80°C until use. Total protein concentration was determined using a Bicinchoninic Acid (BCA) assay (Pierce™ BCA Protein Assay Kits, Thermo Scientific, Waltham, MA, USA). Samples were mixed with 1 volume of 2x Laemmli sample buffer (125 mM Tris–HCl pH 6.8, 20% [v/v] glycerol, 4% [w/v] SDS, 10% [v/v] β‐mercaptoethanol, and 0.005% [v/v] bromophenol blue) and incubated at 65°C for 20 min, followed by incubation on ice for 2 min. Proteins were separated by SDS‐PAGE and transferred to a 0.1‐μm nitrocellulose membrane (10 600 005, Amersham™ Protran™) using a Power Blotter (Invitrogen). Unless noted otherwise, 10 μg total protein was loaded on each gel lane. The membranes were incubated with 3% (w/v) non‐fat dried milk in 1× PBST (137 mM NaCl, 2.7 mM KCl, 10 mM Na_2_HPO_4_, 2 mM K_2_HPO_4_, and 1% [v/v] Tween‐20) for 1 h. The membranes were then incubated in the primary antibody: anti‐HA (1:10000, H6908, Sigma Aldrich), anti‐ZNG3 (1:1000), anti‐ZCP2 (15 000, Agrisera AS12 1848), anti CF_1_ (1:40000, a gift from Sabeeha Merchant) in 3% (w/v) non‐fat dried milk in PBST with constant agitation. The membranes were then washed three times with PBST and incubated with a goat anti‐rabbit secondary antibody conjugated to alkaline phosphatase diluted at 1:8000 (v/v) in 3% (w/v) non‐fat dried milk in PBST for 1 h with constant agitation. Alkaline phosphatase activity was processed according to the manufacturer's instructions.

### RNA extraction and RT‐qPCR

A total of 5–8 ×10^7^ cells were collected by centrifugation at 1610 ***g*** for 2 min at 4°C. The cell pellet was resuspended in 1 mL TRIzol® Reagent (Invitrogen). Then, 200 μL of chloroform was added to each sample, followed by mixing and centrifugation at 13200 rpm (Eppendorf, 5430 R) for 15 min at 4°C. Five hundred microliters of the supernatant was transferred to a new tube containing 700 μL of isopropanol. The mixture was incubated on ice for 15 min, followed by centrifugation at 12 000 rpm (Eppendorf, 5430 R) for 15 min at 4°C. The resulting pellet was dried and resuspended in 40 μL RNase‐free water. Genomic DNA removal and RNA clean‐up were carried out with a Zymo Clean & Concentrator 5 kit (Zymo Research, Irvine, CA, USA) according to the manufacturer's instructions. First‐strand cDNA was synthesized with M‐MLV Reverse Transcriptase (Invitrogen, Carlsbad, CA, USA) and oligo(dT). Quantitative PCR (qPCR) was performed using SYBR Green MasterMix. *RACK1* served as a loading control. Sequences of the *CAH4* primers used for RT‐qPCR are listed in Table S1. The PCR conditions were as follows; 95°C for 10 min, followed by 40 cycles at 95°C for 15 sec and 65°C for 1 min, 95°C for 15 sec, 60°C for 1 min. A melting curve was then generated by raising the temperature to 95°C at a rate of 1°C/sec, before returning to 60°C and held for 1 min.

### RNA sequencing

Two independent wt controls and the two *zng3* mutants were grown phototrophically in HSM medium with either air level of CO_2_ (0.04% [v/v], LC) or high CO_2_ (5% [v/v] CO_2_, HC), or photoheterotrophically in TAP medium with acetate (Ac) as the only reduced carbon source. Each genotype was grown in experimental triplicates for each growth condition, for a total of 36 samples. Total RNA was extracted as described above, abundance was determined using a Qubit RNA BR Assay Kit. Sequencing libraries were prepared using a Watchmaker Genomics mRNA Library Preparation Kit with IDT xGEN 10‐nt Unique Dual‐Index primers following the manufacturer's recommendations. Completed libraries were quantified using a combination of Biotium AccuGreen High Sensitivity dsDNA and Agilent 4200 TapeStation HS DNA1000 assays. Libraries were normalized to a consistent concentration and equal amounts were pooled. The pool titer was quantified using an Invitrogen Collibri Quantification qPCR kit. Sequencing was performed using an Element AVITI Cloudbreak Freestyle High Output 300 cycle kit, as 150‐bp paired‐end reads. Base calling was conducted with AVITIOS v3.3.2; the output was then demultiplexed and converted to FastQ format using Element Biosciences bases2fastq v2.1.0.

Reads were aligned to the most recent version of the *Chlamydomonas* genome (v6) (Craig et al., 2023) using STAR (2.7.11b) (Dobin et al., 2013). Expression estimates in fragments per kilobase of transcript per million fragments mapped reads (FPKM) were determined by Cufflinks and differential expression analysis was performed with Cuffdiff (v2.2.1) (Trapnell et al., 2012). Genes were considered differentially expressed based on the criteria absolute (log_2_ fold‐change) >1 and a false discovery rate (FDR)‐adjusted *P*‐value <0.05. Subsequent data analysis and figure production were carried out in R, and Adobe Illustrator was used for panel assembly.

### Immunoprecipitation followed by tandem mass spectrometry (IP‐MS/MS)

Approximately, 1 × 10^9^ cells were collected by centrifugation at 1610 ***g*** for 3 min at 4°C. The cell pellets were washed twice with 15 mL KH buffer (20 mM HEPES‐KOH pH 7.2, 80 mM KCl) before being resuspended in 1 mL lysis buffer (20 mM HEPES‐KOH pH 7.2, 1 mM MgCl_2_, 1 mM KCl, 15 mM NaCl, 1× protease inhibitor cocktail [Roche], and 0.1% [v/v] Triton X‐100). Cells were lysed by sonication on ice (MISONIX sonicator 3000). Cell debris were removed by centrifugation at 1610 ***g*** for 3 min at 4°C. The supernatant was transferred to a new 1.5‐mL centrifuge tube, to which 5 mg Protein A Sepharose beads (Sigma, P3391) coupled with anti‐HA antibodies (H6908, Sigma Aldrich) were added. The protein–bead mixture was incubated under constant rotation at 4°C for 2 h. The beads were then recovered by centrifugation at 5000 rpm for 20 sec at 4°C, washed three times with lysis buffer (20 mM HEPES‐KOH pH 7.2, 1 mM MgCl_2_, 1 mM KCl, 15 mM NaCl, 1x Protease Inhibitor Cocktail, 0.1% [v/v] Triton X‐100) and twice with 10 mM Tris–HCl pH 7.6. The supernatant was removed, and the beads were frozen for subsequent protein identification by mass spectrometry (MS). For proteolytic digestion, antibody‐bound proteins were digested on‐bead by washing them three times with 50 mM ammonium bicarbonate. Trypsin, resuspended in the same buffer, was then added to the beads at 5 ng/μL to just submerge the beads in digestion buffer for incubation at 37°C for 6 h. The solution was acidified by the addition of 1% trifluoroacetic acid (TFA) and centrifuged at 14000 ***g***. The supernatant containing the digested peptides was removed and concentrated by solid phase extraction using StageTips (Rappsilber et al., 2007). Purified peptide eluates were dried by vacuum centrifugation and frozen at −20°C or resuspended in 2% (v/v) acetonitrile/0.1% (w/v) TFA to a final volume of 20 μL. For LC–MS/MS analysis, an injection of 10 μL was automatically made using a Thermo (www.thermo.com) EASYnLC 1200 system onto a Thermo Acclaim PepMap RSLC 0.1 mm × 20 mm C18 trapping column, followed by a approximately 5‐min wash with buffer A (1% [v/v] formic acid in water). Bound peptides were then eluted over 35 min onto a Thermo Acclaim PepMap RSLC 0.075 mm × 250 mm resolving column at a constant flow rate of 300 nL/min with a gradient of 8–22% buffer B (80% acetonitrile, 19.9% water, 0.1% formic acid, v/v/v) from 0 to 19 min, and 22–40% buffer B from 19 to 24 min. At the end of the gradient, the column was washed with 90% buffer B for the duration of the run. Column temperature was maintained at 50°C using an integrated column oven (PRSO‐V2, Sonation GmbH, Biberach, Germany). Eluted peptides were sprayed into a ThermoScientific Q‐Exactive HF‐X mass spectrometer (www.thermo.com) using a FlexSpray spray ion source. Survey scans were taken in an Orbi trap (60 000 resolution, determined at *m/z* 200) and the top 15 ions in each survey scan were then subjected to automatic higher energy collision induced dissociation (HCD) with fragment spectra acquired at a resolution of 7500. The resulting MS/MS spectra were converted to peak lists using Mascot Distiller, v2.8.5 (www.matrixscience.com) and searched against a protein sequence database based on *Chlamydomonas* genome (v6) (Craig et al., 2023) appended with common laboratory contaminants (downloaded from www.thegpm.org, cRAP project) using the Mascot searching algorithm v 2.8.3. (Perkins et al., 1999) The Mascot output was then analyzed using Scaffold, v5.3.3 (www.proteomesoftware.com) to validate protein identification. Assignments validated using a scaffold 1% FDR confidence filter were considered true. Mascot parameters for all databases were as follows: up to two missed tryptic sites, variable modification of oxidation of methionine, peptide tolerance of ±10 ppm, MS/MS tolerance of 0.02 Da, FDR calculated using randomized database search.

### Statistical analysis

Unless stated otherwise, a two‐sided *t*‐test was used to determine statistical differences between samples. *P*‐values were adjusted to correct for multiple testing using the Benjamini–Hochberg method (Benjamini & Hochberg, 1995). Asterisks between samples indicate an adjusted *P*‐value of <0.05.

## AUTHOR CONTRIBUTIONS

DS and SS designed the research; GK‐A, SS, SCS, AM performed research; GK‐A, SS, TVO, and DS analyzed and interpreted data; DS and SS wrote the paper; authors edited and approved the final version of the manuscript.

## CONFLICT OF INTEREST STATEMENT

The authors declare no conflict of interest.

## Supporting information


**Figure S1.** Fe and Mn contents are similar in wild‐type and cia5 cells grown under LC or HC conditions and in the presence of acetate. Wild‐type (wt) and *cia5* mutants were grown phototrophically under high CO_2_ (HC) or low CO_2_ (LC) conditions, or photoheterotrophically (with acetate [Ac] as sole reduced carbon source). (a) Cell size distribution of all genotypes across growth conditions, determined using a Z2 Coulter Counter. The horizontal bars indicate the median values from four or more independent experiments (*n* > 3). Data from individual cells are also shown. (b, c) Iron (Fe, b) and manganese (Mn, c) content in the indicated genotypes under each growth condition, determined by ICP‐MS/MS and normalized to cell number. Values are means ± standard deviation (SD) from six independent experiments. Individual data points are also shown as open circles.
**Figure S2.** Multiple sequence alignment of CobW (COG0532) domain proteins from *Chlamydomonas reinhardtii*, *Arabidopsis thaliana*, and *Saccharomyces cerevisiae*. Sequences were aligned using Clustal Omega (https://pubmed.ncbi.nlm.nih.gov/21988835/) and organized in Jalview (https://doi.org/10.1093/bioinformatics/btp033). The CobW domain (N‐terminal, GTPase) is indicated by a horizontal orange bar above the protein sequences, the C‐terminal CobW_C domain is indicated by a red bar above the sequences. Important motifs are highlighted by a black outline and labeled accordingly. *Chlamydomonas* CobW proteins are labeled green on the tree to the left, with the exception of ZNG3, which is labeled in black and its amino acid sequence is highlighted by a gray background. Arabidopsis proteins are labeled in blue, *Saccharomyces cerevisiae* ZNG1 is labeled in gray.
**Figure S3.** Transcript abundance of genes encoding Zn importers and Zn chaperones in response to Zn deficiency, CO_2_ supply, and along the diurnal cycle. Survey of transcript abundance for putative Zn transporter genes and candidate chaperone genes in published RNA‐seq datasets with varying Zn and CO_2_ supply. (1) Expression in phototrophically grown cultures along a diurnal cycle (12‐h dark/12‐h light) (Strenkert et al., 2019); (2) Zn‐replete (+) and Zn‐deficient (−) cultures (Malasarn et al., 2013); (3) Early exponential and early stationary Zn‐replete cultures (+), as well as Zn‐deficient (−) cultures and Zn resupply (Hong‐Hermesdorf et al., 2014); (4) Cultures acclimated to high (+, 5%), air level (=, 0.04%) and low CO_2_ (−, 0.01%) in wild‐type (black outline) and a *cia5* mutant (red outline) (Fang et al., 2012); (5) Transition from high (+, 5%) to very low CO_2_ supply (−, 0.01%) (Brueggeman et al., 2012).
**Figure S4.** Expression profiles of genes involved in the CCM and CO_2_ assimilation. Heatmap representation of transcript levels (left, in FPKM) and log_2_ fold‐changes (right) for genes encoding proteins involved in the CCM and CO_2_ assimilation between *zng3* mutants and wild‐type.
**Figure S5.** Expression of genes involved in Zn assimilation and distribution. Heatmap representation of transcript abundance for genes encoding proteins involved in Zn assimilation and distribution. The heatmap on the left highlights transcript abundance in FPKM, the heatmap on the right shows the log_2_ fold‐changes between *zng3* and wt.
**Figure S6.** Expression of genes involved in protein quality control. Heatmap representation of transcript abundance for genes encoding proteins involved in protein quality control. The heatmap on the left highlights transcript abundance in FPKM, the heatmap on the right shows the log_2_ fold‐change between *zng3* and wt.

**Table S1.** List and sequences of sgRNAs, primers, and ssODNs used in this study.

**Data S1.** List of CIA5‐interacting proteins identified by immunoprecipitation followed by mass spectrometry (IP‐MS/MS) that met filtering criteria.

**Data S2.** List of ZNG3‐interacting proteins identified by IP‐MS/MS that met filtering criteria.

**Data S3.** Transcript abundance data estimated in FPKM from wild‐type (wt) and *zng3* mutants cultured photoheterotrophically (acetate, Ac) or phototrophically with bubbling with 5% CO_2_ (HC) or air (LC).

## Data Availability

The authors confirm that the data supporting the findings of this study are available within the article and its supplementary files. Raw data that support the findings of this study are available from the corresponding author upon reasonable request. Transcriptome data were deposited in the NCBI Sequence Read Archive (SRA), under accession number PRJNA1308488.
