# Supplementary material for: CIA5 and its interacting metal‐binding GTPase ZNG3 are degraded by the proteasome in Zn deficiency
Source: Plant J. 2026 Jul 14;127(1):e71035. doi: 10.1111/tpj.71035 (PMC13368412; doi:10.1111/tpj.71035)
Supplement: Supplementary file 1 — Figure S1. Fe and Mn contents are similar in wild‐type and cia5 cells grown under LC or HC conditions and in the presence of acetate. Wild‐type (wt) and cia5 mutants were grown phototrophically under high CO2 (HC) or low CO2 (LC) conditions, or photoheterotrophically (with acetate [Ac] as sole reduced carbon source). (a) Cell size distribution of all genotypes across growth conditions, determined using a Z2 Coulter Counter. The horizontal bars indicate the median values from four or more independent experiments (n > 3). Data from individual cells are also shown. (b, c) Iron (Fe, b) and manganese (Mn, c) content in the indicated genotypes under each growth condition, determined by ICP‐MS/MS and normalized to cell number. Values are means ± standard deviation (SD) from six independent experiments. Individual data points are also shown as open circles. Figure S2. Multiple sequence alignment of CobW (COG0532) domain proteins from Chlamydomonas reinhardtii, Arabidopsis thaliana, and Saccharomyces cerevisiae. Sequences were aligned using Clustal Omega (https://pubmed.ncbi.nlm.nih.gov/21988835/) and organized in Jalview (https://doi.org/10.1093/bioinformatics/btp033). The CobW domain (N‐terminal, GTPase) is indicated by a horizontal orange bar above the protein sequences, the C‐terminal CobW_C domain is indicated by a red bar above the sequences. Important motifs are highlighted by a black outline and labeled accordingly. Chlamydomonas CobW proteins are labeled green on the tree to the left, with the exception of ZNG3, which is labeled in black and its amino acid sequence is highlighted by a gray background. Arabidopsis proteins are labeled in blue, Saccharomyces cerevisiae ZNG1 is labeled in gray. Figure S3. Transcript abundance of genes encoding Zn importers and Zn chaperones in response to Zn deficiency, CO2 supply, and along the diurnal cycle. Survey of transcript abundance for putative Zn transporter genes and candidate chaperone genes in published RNA‐seq datasets wi [file TPJ-127-0-s004.docx]

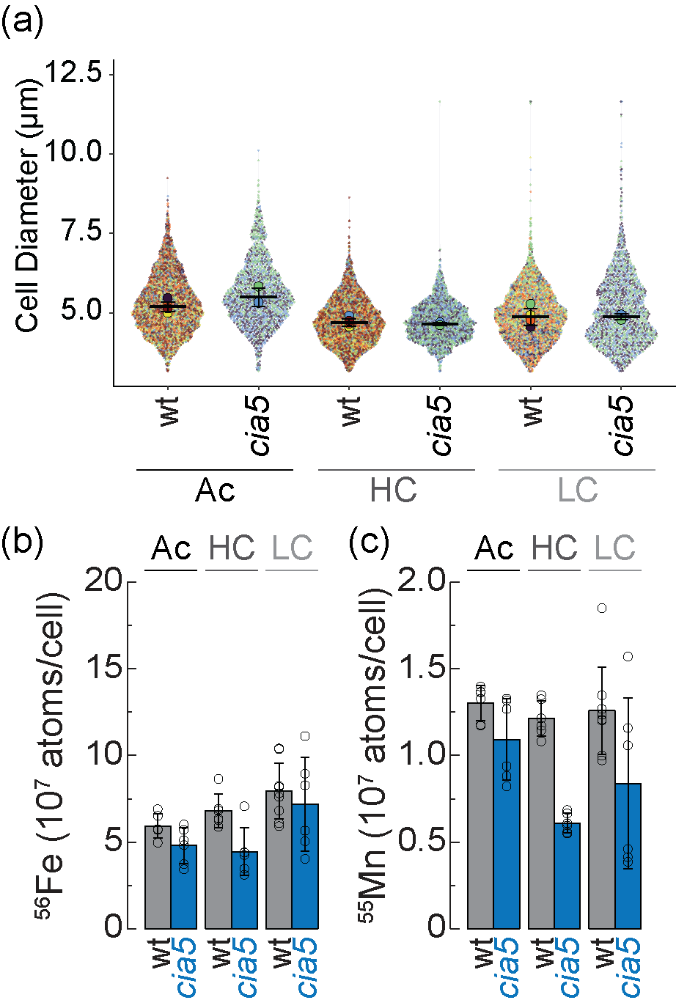


**Supplemental Figure 1. Fe and Mn contents are similar in wild-type and cia5 cells grown under LC or HC conditions and in the presence of acetate.** Wild type (wt) and *cia5* mutants were grown phototrophically under high CO_2_ (HC) or low CO_2_ (LC) conditions, or photoheterotrophically (with acetate [Ac] as sole reduced carbon source). **(a)** Cell size distribution of all genotypes across growth conditions, determined using a Z2 Coulter Counter. The horizontal bars indicate the median values from four or more independent experiments (*n*>3). Data from individual cells are also shown. **(b,c)** Iron (Fe, **b**) and manganese (Mn, **c**) content in the indicated genotypes under each growth condition, determined by ICP–MS/MS and normalized to cell number. Values are means ± standard deviation (SD) from six independent experiments. Individual data points are also shown as open circles.


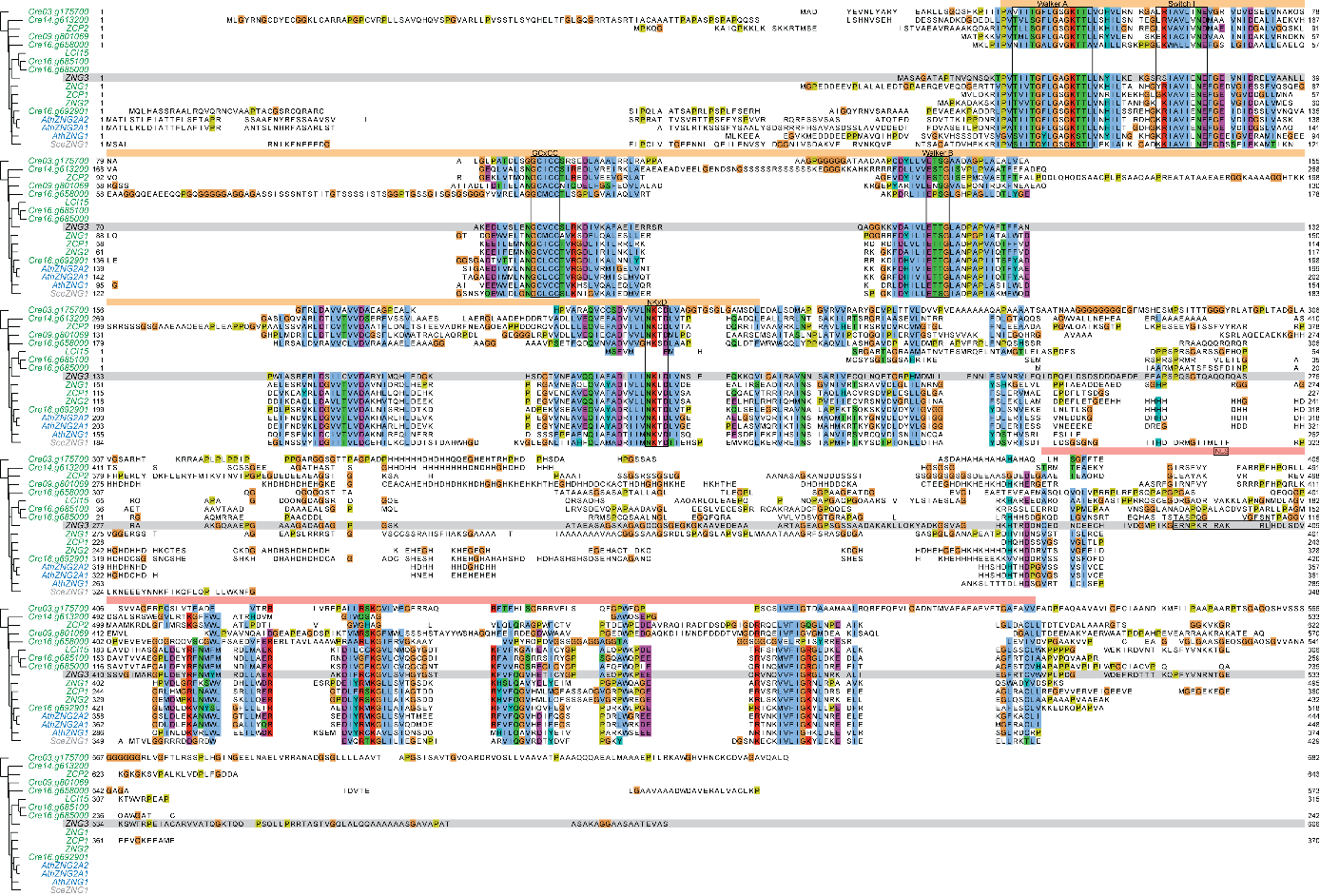


**Supplemental Figure 2. Multiple sequence alignment of CobW (COG0532) domain proteins from *Chlamydomonas reinhardtii*, *Arabidopsis thaliana*, and *Saccharomyces cerevisiae*.** Sequences were aligned using Clustal Omega (<https://pubmed.ncbi.nlm.nih.gov/21988835/>) and organized in Jalview (<https://doi.org/10.1093/bioinformatics/btp033>). The CobW domain (N-terminal, GTPase) is indicated by a horizontal orange bar above the protein sequences, the C-terminal CobW_C domain is indicated by a red bar above the sequences. Important motifs are highlighted by a black outline and labeled accordingly. Chlamydomonas CobW proteins are labeled green on the tree to the left, with the exception of ZNG3, which is labeled in black and its amino acid sequence is highlighted by a gray background. Arabidopsis proteins are labeled in blue, *Saccharomyces cerevisiae* ZNG1 is labeled in gray.

**
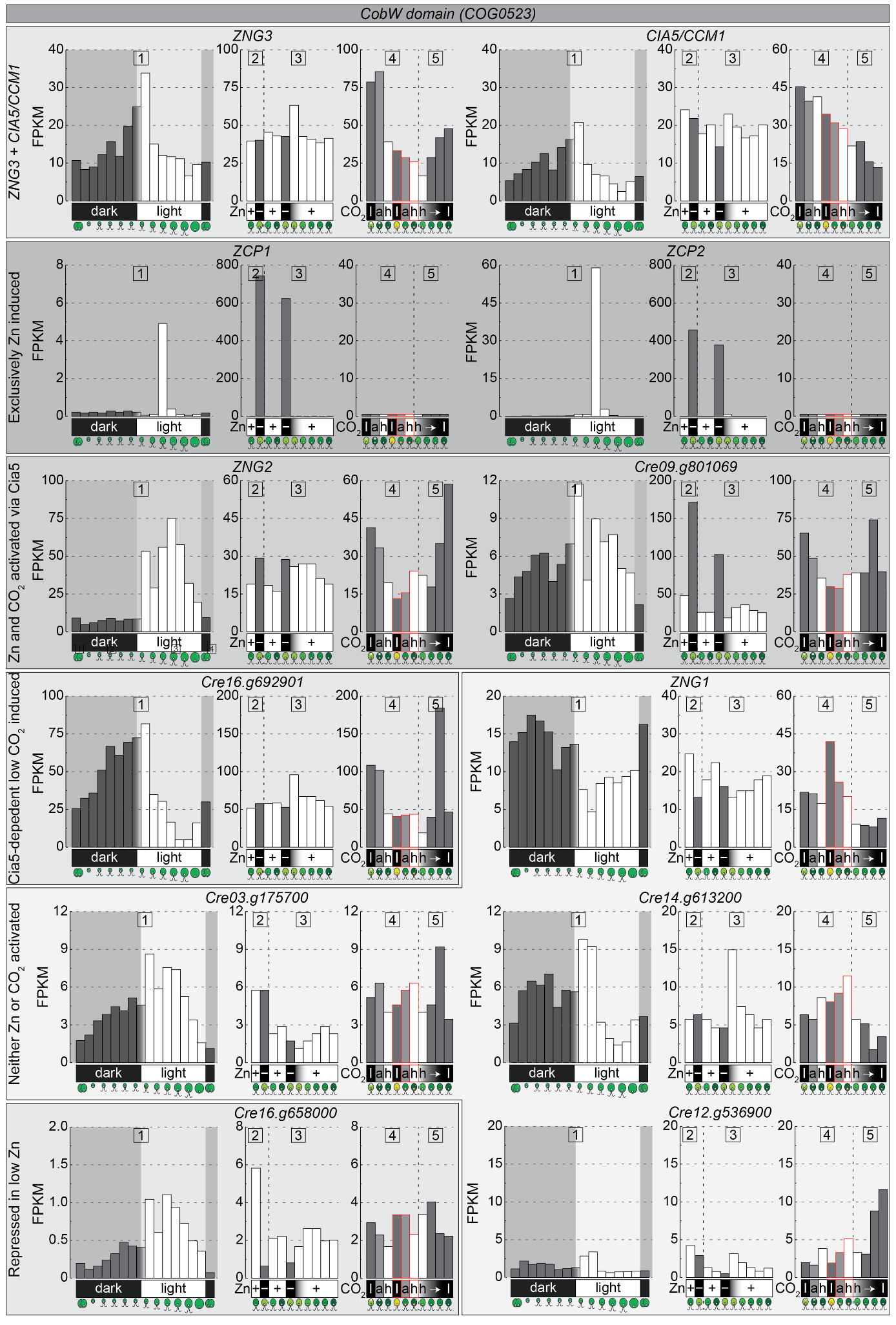
**


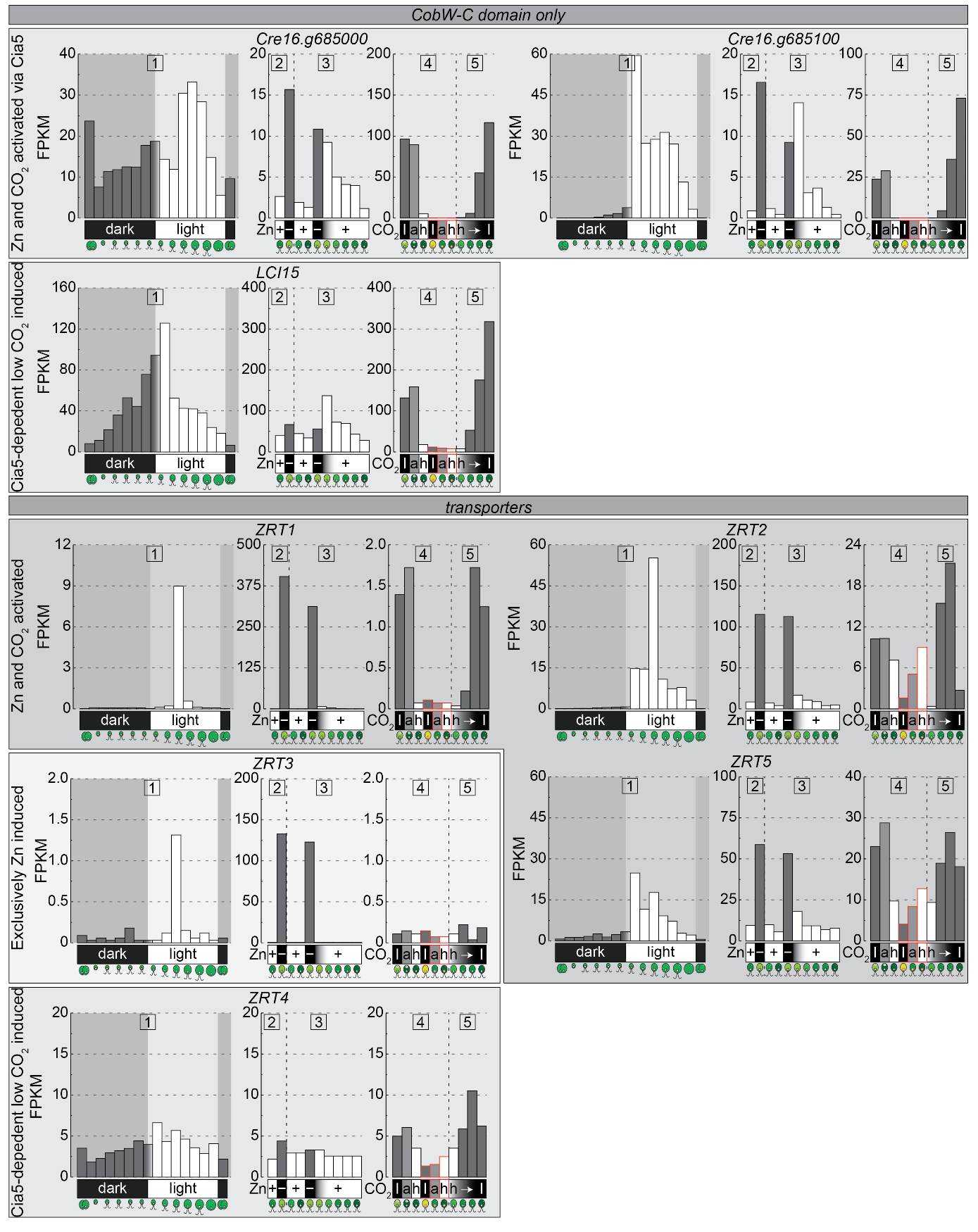
 **Supplemental Figure 3. Transcript abundance of genes encoding Zn importers and Zn chaperones in response to Zn deficiency, CO_2_ supply, and along the diurnal cycle.** Survey of transcript abundance for putative Zn transporter genes and candidate chaperone genes in published RNA-seq datasets with varying Zn and CO_2_ supply. (1) Expression in phototrophically grown cultures along a diurnal cycle (12-h dark/12-h light) (Strenkert *et al.,* 2019); (2) Zn-replete (+) and Zn-deficient (−) cultures (Malasarn *et al.,* 2013); (3) Early exponential and early stationary Zn-replete cultures (+), as well as Zn-deficient (−) cultures and Zn resupply (Hong Hermesdorf *et al.,* 2014); (4) Cultures acclimated to high (+, 5%), air-level (=, 0.04%) and low CO_2_ (−, 0.01%) in wild type (black outline) and a *cia5* mutant (red outline) (Fang *et al.,* 2012); (5) Transition from high (+, 5%) to very low CO_2_ supply (−, 0.01%) (Brueggemann *et al.,* 2012).

**
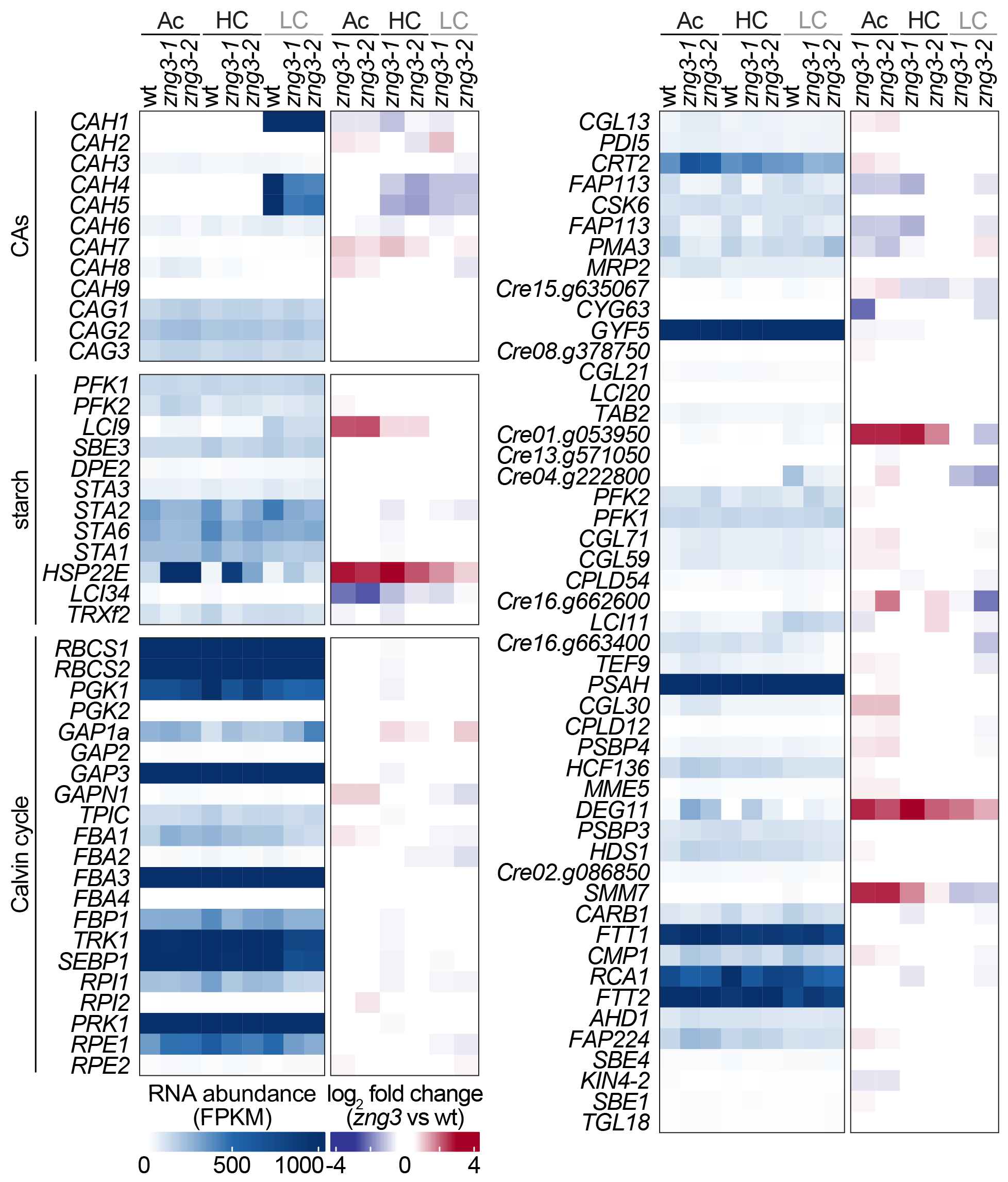
 Supplemental Figure 4. Expression profiles of genes involved in the CCM and CO_2_ assimilation.** Heatmap representation of transcript levels (left, in FPKM) and log_2_ fold-changes (right) for genes encoding proteins involved in the CCM and CO_2_ assimilation between *zng3* mutants and wild type.

**
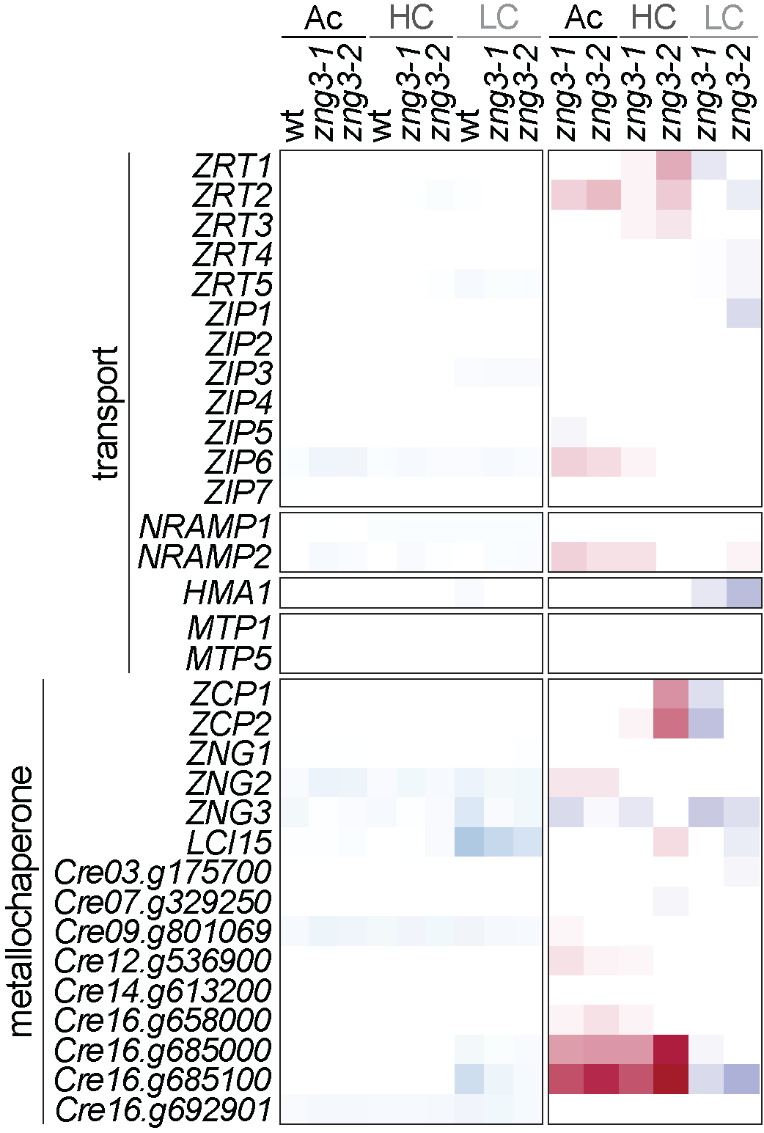
**

**Supplemental Figure 5. Expression of genes involved in Zn assimilation and distribution.** Heatmap representation of transcript abundance for genes encoding proteins involved in Zn assimilation and distribution. The heatmap on the left highlights transcript abundance in FPKM, the heatmap on the right shows the log_2_ fold-changes between *zng3* and wt.

**
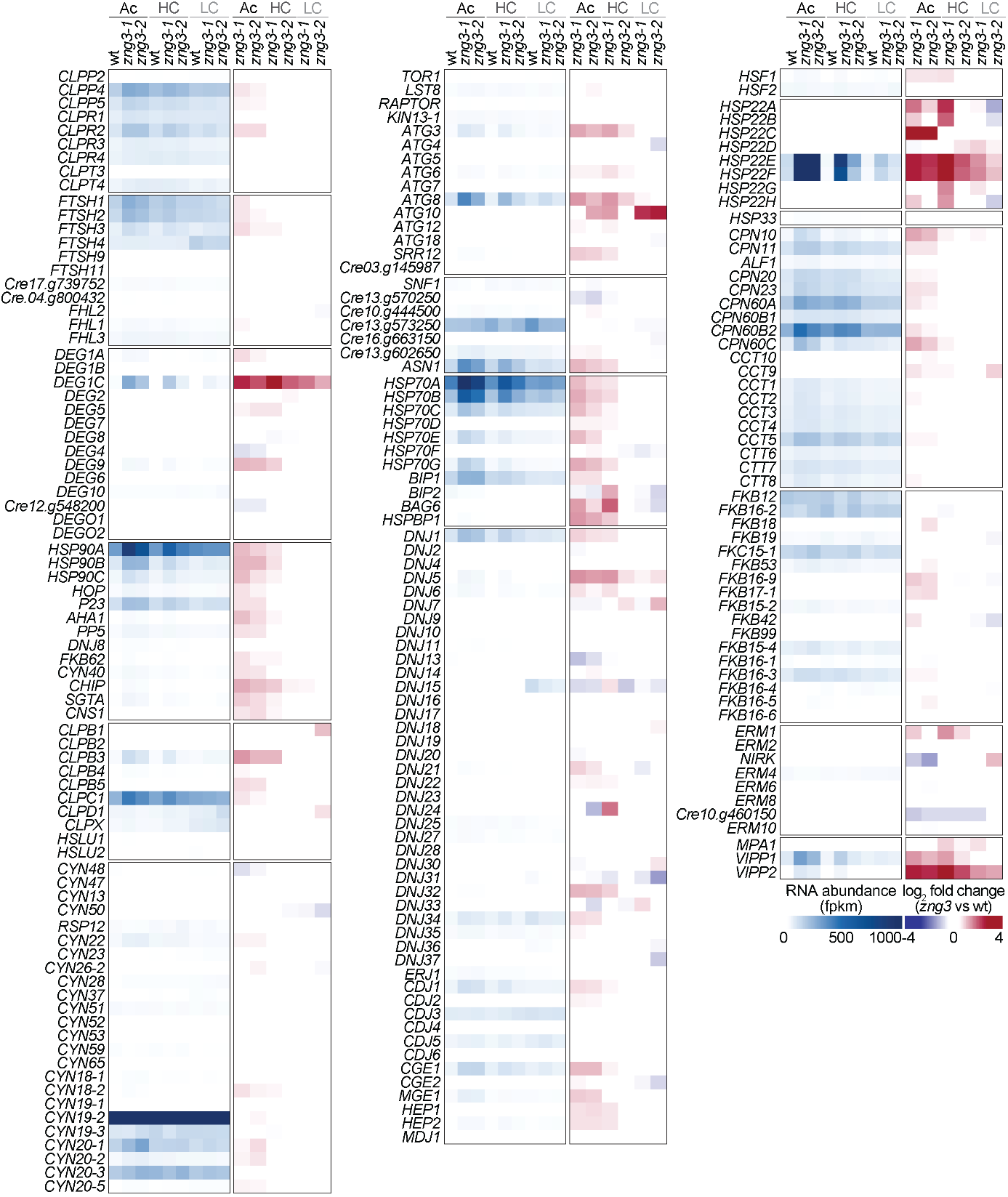
 Supplemental Figure 6. Expression of genes involved in protein quality control.** Heatmap representation of transcript abundance for genes encoding proteins involved in protein quality control. The heatmap on the left highlights transcript abundance in FPKM, the heatmap on the right shows the log_2_ fold-change between *zng3* and wt.
