# Supplementary material for: CIA5 and its interacting metal‐binding GTPase ZNG3 are degraded by the proteasome in Zn deficiency
Source: Plant J. 2026 Jul 14;127(1):e71035. doi: 10.1111/tpj.71035 (PMC13368412; doi:10.1111/tpj.71035)
Supplement: Supplementary file 2 — Table S1. List and sequences of sgRNAs, primers, and ssODNs used in this study. [file TPJ-127-0-s002.docx]

**Supplemental table 1.** Sequences of gRNAs, primers and ssODNs used in this study.

|  | **Function** | **Name** | **Sequence 5’-3’** |
| --- | --- | --- | --- |
| Generation of *cia5-1* null mutant | Guide RNA | CIA5 gRNA1 | TTTCGCGGAGCTATGGCGCCTAAAG |
|  | ssODN | CIA5 ssODN1 | TGGTCCACGCTGTGTTGGTCGCAGTTACCCCGGCTGCAATCGGAGTTTCGCGTAATAGTGGCGCCTAAAGGTCCATTATCGAGCTCCTCCGGACATTCGGGGGAGTGGGAAGGAGCGAGG |
|  | Primer | CIA5screenfor1 | GCAATCGGAGTTTCGCGTAATAG |
|  | Primer | CIA5screenrev1 | CATTTCGGGCAGTGGGTGAG |
| Generation of *cia5-2* null mutant | Guide RNA | CIA5 gRNA2 | GTTTCGCGGAGCTATGGCGCCTAAA |
|  | ssODN | CIA5 ssODN1 | TATTTGGCAGGCTTTGCGGACAGGAGAGGGCTAACCTTGGCTGTGCGCTGGTCCACGCTGTGTTGGTCGCAGTTACCCCGGCTGCAATCGGAGTTAAGCGTAACTATGGCGCCTAAAGGTCCATTATCGAGCTCCTCCGGACATTCGGGGGAGTGGGAAGGAGCGAGGCCACGGAACGGAGCTCACCCACTGCCCGAA |
|  | Primer | CIA5screenfor2 | CTGCAATCGGAGTTAAGCGTAA |
|  | Primer | CIA5screenrev2 | GCCGGCAAGTGATAGGCTAA |
|  | Primer | CIA5seqfor | AACGCTTCTATTGCCCGTGA |
|  | Primer | CIA5seqrev | AAGAAGCAAGACGCCCAGAG |
| Generation of Cia5-HA strains | Guide RNA | CIA5HA gRNA | TTTGCCATCACTGATCGATGTCTGT |
|  | ssODN | CIA5HA ssODN | GCACCGTCGCAGCTGTACTTGACGGCCAGGGTTTGCCATCACTGATCGATGTCTGTGTTGTGCTTAAACTCGGCGCCATGCAGGGACCACTTGTTGGATGCGGAGACGTTCCGACTGCTGCAGTCCTGCGATggcggtggcTACCCGTACGACGTGCCCGATTACGCTTAGGCGGCGAGACGAGCCCTGGCTGCGTGCCA |
|  | Primer | CIA5HAseqfor | ACCGTCGCAGCTGTACTTGAC |
|  | Primer | CIA5HAseqrev | ATCATACGCGACACGCACTTG |
| Generation of ZNG3-HA strains | Guide RNA | ZNG3HA gRNA | TAGCGTCCTAGTCCATCGACTGG |
|  | ssODN | ZNG3HA ssODN | CAGCGACGGCGTCGGCCAAAGCAGGGGGCGCAGCCTCAGCAGCAACGGAGGTAGCGTCCggcggtggcTACCCGTACGACGTGCCCGATTACGCTTAGTCCATCGACTGGAGCCGGTATCGCGACACCCTCACTGATCCGCTTGGCGA |
|  | Primer | ZNG3HAscreenfor2 | CCTCGGGTCCTGGCCATACA |
|  | Primer | ZNG3HAscreenrev2 | GCACGTCGTACGGGTAGCCAC |
|  | Primer | ZNG3HAseqfor | CCGTTCTACGTGAACCGCAA |
|  | Primer | ZNG3HAseqrev | AACTGGCGCTGAACAAGCAG |
| Generation of *zng3* mutant strains | Guide RNA | ZNG3 gRNA | TTTGAGCTAGGGGCATTTCGCCAGA |
|  | ssODN | ZNG3 ssODN | GGCCAGCAAGCGCAGTACACTGGTCGGCTTCATAAACAATAAACCTGTTCAATGATACTGACCCGATTTCCTTTGAGCTAGGGGCATTTCGCCAGAGAGCTGTCGCATAGATCGCCTTTGCGCTCGCAACTCCCCGTTGCTTTTGAGCCCTCGCCGCCCTCTGCGCCCTCCTCGCTGTAACGCAAGGTCAGTGTTTGTTTTTCACACCGTGACCGTGGGTGAGCATGCAGTTGGCTCTGCGCCTGGGGCTTTGCGTGCAACGGGACCAGGAATTGCGTCTGCACCCAGTTGTGGGCGTTGTGGGCGTTGCGTTGCATACGGGCATGTGGGCGCGCGTCGCATGGTCTTGTGTTGTGTCCATAGTCACAATTGGCATGGGGCGCGGGCGCGTTAGGCTGCATGAGGGTGCTAGGCTGGTTACGGCCCGGTACCACATTGCCCAGGCGCTTGCACTCGCACTCGCACATGTTCTTGTTCATGCTAACAACCGGCCGCACGGCCTCGGATCACCAGGGGTGCTCCCACCATGTATTCGCACAGACTCGCGCGCTCGCGGCCGGCATTGCCGGCCCGGGACGTGATCTTGCGTTGCATTGCGGGCAGCTGAGAAAGACCCTCACCACCGGAACGAGATAATGCTCGCGTCGGGAGTGTGTTTCCGTGCCTGCGCGTGCGTGCGTGCGGCGCCTGGCGCGGCTGACACCCTTCCCATGGGTCGGCCGCGTCTGGCGTCCGCCGCCGCCGTCGCCTTCCCCCCCTCTCCCATCTCGCCGGTGTCATCTTCAGACTCGACATTGCTAATTGGCATCGGCTTCTCTCGCTCTCTGGCGACGACTGCTGCGGCGCTGGCCTTATCATTCGGGCATGTCACTGACGGTAAGTGAGAAGGCTTCGGTTTTGTGCCGTCAACTTCTAGGGTGTCTGCGCTACTGCGTAAGATGCCGCCCTGCCTGCCTGTGTCCAGCGCTGGCTTCTTCCGCCCATCCGCAACCCGCCCCTCATCCTGCCTGCTTGCGGTGCTTGCGCGCGCATTGGGGTCTCCTCCTCCCCCTCCCCTCTCTCCTCACCCCTTCCCGGGCCACGCTGCCTCCGTGCAATGCGTTGGGCGGCACACAGCCCCTCGCATCGGCCCGCGCCCGCGCTGCTCGCCCGCCCGCCTCCTCCCCCCTGCCCCTCCTTTCTCAACCTTCCAGAACCTTCTTCACCAAAGATGGCCAGCGCCGGTGCCACCGCCCCGACAAACGTTCAGAACTCACAAAAGACGCCTGTCACCATCATCACTGGCTTTCTAGGGGTGAGACCAGGACTCGATAGCTGCTC |
|  | Primer | ZNG3 seqfor1 | GGCCAGCAAGCGCAGTACA |
|  | Primer | ZNG3 seqrev1 | AGTGATGATGGTGACAGGCGT |
| qRT-PCR | Primer | CAH4For | TTCGCCGCCTTCCTGTCCTG |
|  | Primer | CAH4Rev | GTCACGATGTTGCCGGCCAC |
| Generation of *cia5 zng3* double mutants in *zng3-1* | Guide RNA | CIA5 gRNA2 | GTTTCGCGGAGCTATGGCGCCTAAA |
|  | ssODN | CIA5 ssODN2 | TATTTGGCAGGCTTTGCGGACAGGAGAGGGCTAACCTTGGCTGTGCGCTGGTCCACGCTGTGTTGGTCGCAGTTACCCCGGCTGCAATCGGAGTTAAGCGTAACTATGGCGCCTAAAGGTCCATTATCGAGCTCCTCCGGACATTCGGGGGAGTGGGAAGGAGCGAGGCCACGGAACGGAGCTCACCCACTGCCCGAA |
|  | Primer | CIA5 screenfor2 | CTGCAATCGGAGTTAAGCGTAA |
|  | Primer | CIA5 screenrev2 | GCCGGCAAGTGATAGGCTAA |
|  | Primer | CCM1 seqfor2 | AACGCTTCTATTGCCCGTGA |
|  | Primer | CCM1 seqrev2 | AAGAAGCAAGACGCCCAGAG |
| Generation of *cia5 zng3* double mutants in *zng3-2* | Guide RNA | CIA5 gRNA2 | GTTTCGCGGAGCTATGGCGCCTAAA |
|  | ssODN | CIA5 ssODN3 | CGCTGGTCCACGCTGTGTTGGTCGCAGTTACCCCGGCTGCAATCGGAGTTAGGCGTAACTATGGCGCCTAAAGGTCCATTATCGAGCTCCTCCGGACATTCGGGGGAGTGGGAAGGAGCGAG |
|  | Primer | CIA5 screenfor3 | CTGCAATCGGAGTTAGGCGTAA |
|  | Primer | CIA5 screenrev3 | GCCGGCAAGTGATAGGCTAA |
|  | Primer | CIA5 seqfor2 | AACGCTTCTATTGCCCGTGA |
|  | Primer | CIA5 seqrev2 | AAGAAGCAAGACGCCCAGAG |
